# Sexual health and healthy relationships for Further Education (SaFE) in Wales and England: results from a pilot cluster randomised controlled trial

**DOI:** 10.1136/bmjopen-2024-091355

**Published:** 2024-12-20

**Authors:** Rhys Williams-Thomas, Julia Townson, Ruth Lewis, Lauren Copeland, Jason Madan, G J Melendez-Torres, Fiona V Lugg-Widger, Philip Pallmann, Muhammad Riaz, Rachel Brown, Chris Bonell, Gemma S Morgan, James White, Honor Young

**Affiliations:** 1 Centre for Trials Research, Cardiff University College of Biomedical and Life Sciences, Cardiff, UK; 2 Institute of Health and Wellbeing, MRC Social and Public Health Sciences Unit, Glasgow, UK; 3 Cardiff Metropolitan University, Cardiff, UK; 4 University of Warwick, Warwick Medical School, Coventry, Warwick, UK; 5 Faculty of Health and Life Sciences, University of Exeter, Exeter, UK; 6 Centre for the Development, Evaluation, Complexity and Implementation in Public Health Improvement, Cardiff University School of Social Sciences, Cardiff, UK; 7 Department of Public Health, Environments and Society, London School of Hygiene and Tropical Medicine, London, UK; 8 Public Health and Wellbeing Division, South Gloucestershire Council, Yate, UK

**Keywords:** Health, Nurses, SEXUAL MEDICINE, Schools, Sexually Transmitted Disease

## Abstract

**Objectives:**

To examine the acceptability of implementing, trialling and estimating the cost of the Sexual health and healthy relationships for Further Education (SaFE) intervention.

**Design:**

Two-arm repeated cross-sectional pilot cluster randomised controlled trial (cRCT) of SaFE compared with usual practice, including a process evaluation and an economic assessment.

**Setting:**

Eight further education (FE) settings in South Wales and the West of England, UK.

**Participants:**

FE students, staff and sexual health nurses.

**Intervention:**

SaFE had three components: (1) onsite access to sexual health and relationship services provided by sexual health nurses available for 2 hours on 2 days per week; (2) publicity about onsite sexual health and relationship services and (3) FE staff training on how to promote sexual health, and recognise, prevent and respond to dating and relationship violence (DRV) and sexual harassment.

**Primary and secondary outcome measures:**

The primary outcome was feasibility, assessing whether the study met progression criteria relating to: (a) FE setting and student recruitment; (b) the acceptability of the intervention and (c) qualitative data, and documentary evidence from students, staff and sexual health nurses on acceptability, fidelity of implementation and receipt. We also assessed the completeness of primary, secondary and intermediate outcome measures and estimated cost of the intervention.

**Results:**

Three of the four progression criteria were met. Eight FE settings were recruited, randomised and retained. Of the students approached, 60.7% (1124/1852 students) at baseline and 51.9% (1139/2193 students) at 12 month follow-up completed the questionnaire (target 60%). Over 80% of onsite sexual health services were attended by a nurse; onsite publicity about sexual health services was observed at all intervention settings and 137 staff were trained. SaFE was viewed positively by FE students, FE staff and nurses but needed more time to embed. The prevalence of self-reported unprotected sex at last intercourse was 15.5% at baseline and 18.7% at follow-up. There was evidence of floor effects in the measure of DRV victimisation in the last 12 months. We found low rates of missing data for almost all variables with no discernible differences across arms. The estimated cost per FE setting was £38,363.09.

**Conclusions:**

SaFE was implemented and well received by students, staff and nurses. If strategies to boost student recruitment to the survey can be identified, progression to a phase III effectiveness trial of SaFE is warranted.

**Trial registration number:**

ISRCTN54793810.

**STRENGTHS AND LIMITATIONS OF THIS STUDY**

- This pilot cluster randomised controlled trial used mixed methods to systematically address uncertainties in the acceptability of the intervention and trial design.
- The involvement of FE staff, students and intervention delivery staff in the assessment of feasibility enabled an in-depth exploration of their experiences and views of the intervention.
- Qualitative interviews with students were with a self-selecting sample, so may not represent the views of the wider student population.
- COVID-19 pandemic restrictions at further education settings meant that the intervention was not implemented for as long as planned (up to 23 weeks vs 39 weeks).

## Introduction

 Sexual health includes positive, pleasurable, respectful and safe sexual relationships, and experiences free of coercion, discrimination and violence.^1^ However, many young people’s experiences fall short of this.^2^

Sexually transmitted infections (STIs) are currently at a 10 year high in England^3^ and Wales.^4^ In 2022, young people aged 15–24 accounted for 65% of chlamydia cases, 21% of genital warts, 45% of genital herpes, 57% of gonorrhoea diagnoses and 7% of new HIV diagnoses.^4^ In the UK, 50% of young people attending further education (FE) also report experience of dating or relationship violence (DRV) and among 16–19-year-olds, 46%–50% report controlling behaviours and 27%–32% threatening behaviours.^5^ The median age for most recent occurrence of non-volitional sex is 18 among men and 16 among women.^6^ Education settings are a common environment in which harassment occurs with 67% of girls aged 13–18 years reporting sexual harassment at school or college; 18% experiencing unwanted touching, such as being pinned down or having their bra strap or skirt pulled.^7 8^Early experience of DRV is associated with subsequent adverse outcomes such as STIs and mental health problems.^9 10^

The UK also has one of the highest rates of under-18 births in western Europe,^11^ with 21% of all unplanned pregnancies in 2013 occurring among 16–18 year-olds,^12^ and 53.3% of conceptions in 2021 among under 18s leading to termination of pregnancy.^13^ Even after controlling for prior disadvantage, teenage pregnancy is associated with adverse medical, social, educational and economic outcomes for both mothers^14^,^16^ and children.^17 18^

Systematic reviews suggest that comprehensive interventions addressing sexual health knowledge, contraception availability and broader youth development are most effective at improving sexual health outcomes and preventing teenage conceptions.^19^ Cochrane and Campbell reviews recommend prioritising research on multicomponent interventions in schools.^20^,^22^ They suggest interventions should attempt to improve skills (e.g., conflict management) and shift peer norms against DRV and provide adolescents with school-based health services with a range of contraceptive choices as well as involving young people in the design of services.^23^ In 2022, National Institute for health and Care Excellence (NICE) recommended that sexual health services should be considered in non-clinical settings.^24^ In the UK, FE settings comprise sixth form (often attached to secondary schools) and community colleges where people undertake education and training after secondary education but not part of higher/university education. FE provides a setting for delivering interventions to prevent DRV and improve sexual health in young people. However, FE settings have a transient student population with flexible timetables and attendance is only needed on campus at certain times of the day or week, sites also vary considerably in size as well as range of programmes and services offered.^25^ This poses a considerably different challenge to intervention delivery and evaluation compared with schools.

In response to this gap in the evidence base, we conducted a phase I intervention development study where we co-produced with stakeholders an intervention logic model, theory of change and candidate intervention components of a DRV prevention and sexual health intervention in FE settings.^26^ The study found broad support for two of four components: onsite sexual health and relationship services and FE staff training to prevent and intervene to stop DRV and sexual harassment.^26^ Following this study, we engaged with a wider array of stakeholders including FE students, FE staff and nurses to optimise the staff training on DRV and sexual harassment prevention and advertising of onsite sexual health services. Findings from the optimisation phase and an assessment of willingness to consent to linkage to electronic sexual health records will be reported elsewhere. Here we report the results of a pilot cluster randomised controlled trial (cRCT) examining implementation and acceptability of the intervention and trial methods against progression criteria to understand whether a full-scale evaluation of the intervention is warranted.

## Methods

### Study design and participants

This study involved a two-arm repeated cross-sectional pilot cluster randomised controlled trial (cRCT) of SaFE compared with usual practice, including a process evaluation and an economic assessment. The full details of the study design can be found in our published protocol elsewhere.^27^ This pilot study was reported in accordance with the Consolidated Standards of Reporting Trials (CONSORT) extension for pilot and feasibility trials.^28^ It was conducted in the West of England and South Wales. FE settings were invited to participate, and all interested were visited to discuss the study in more detail and agree a research contract. Eight FE settings were sampled from those wishing to take part to contain: four from each country (England, Wales); four of each type (sixth form, FE college). All state-funded FE settings including community colleges and sixth forms attached to secondary schools were eligible to participate, including private and Welsh-medium schools.

Eligible settings were approached and invited to participate via a relevant senior manager (e.g., deputy head, head of pastoral care), identified with the help of the School Health Research Network (for sixth forms in Wales) and public health leads and service providers in local authorities in England. FE settings were emailed or posted a project information sheet, reply envelope and form indicating their wish to participate. They were followed up by phone call. All interested settings were visited by the SaFE trial manager and a contact from the intervention delivery team to discuss the trial in more detail and agree a research contract describing the roles, responsibilities, timeline of intervention delivery and assessments before taking part.

*Exclusion criteria*: students aged 15 years or below were not eligible to receive services or complete the student survey. Settings exclusively for students with learning disabilities were excluded. Settings with existing onsite sexual health service provision (e.g., STI testing) were excluded from the sampling frame. However, sites with condom provision were permitted.

*Study population*: SaFE is designed to be a universal intervention for all students attending FE settings. The majority of FE students are aged 16–24. FE setting retention was incentivised with a £500 payment on completion of the study.

### Randomisation and blinding

Randomisation occurred after all settings completed baseline data collection. Clusters (settings) were randomised to receive either the SaFE intervention or usual practice. Following baseline surveys (September/October 2021), the trial statistician randomly allocated settings into two arms using a 3:1 ratio: SaFE delivered in six settings and usual practice in two. The unequal allocation ratio was used to gather more information on the acceptability of the intervention. The allocation was stratified by country and type of setting. All parties were blind to allocation during the baseline data collection. It was not possible for study participants (students), FE staff, trial managers, the intervention delivery team or researchers involved in the process evaluation to be blind to intervention status. However, fieldworkers at outcome data collections remained blind to intervention status as was the statistician analysing the primary and secondary outcome data and the health economist undertaking the economic analysis. If school/college allocation became apparent, we recorded this.

### Data collection

Baseline measures were collected in September and October 2021 and assessed via a student self-report survey prior to randomisation. A second set of measurements were taken 12 months postbaseline in September and October 2022, the intervention starting with staff training in November 2021 and the onsite services and publication running January to July 2022. Informed by protocols refined in the MRC-funded SaFE^26^ and NIHR-funded Filter study,^29^ data collections took place during sessions lasting up to 3 hours sessions across 3 days at each FE site. Trained fieldworkers attended social areas and lessons to invite students to participate.

Structured observations of staff training (n=1 per setting), focus groups with students (up to n=2 per setting) and telephone interviews with trained FE staff (up to n=4 per setting) and onsite sexual health service staff (n=1 per setting) examined intervention acceptability, delivery and institutional or student-level barriers to implementation. Logbooks for onsite sexual health service staff examined service provision. Logbooks for FE staff examined time and resources spent implementing the intervention.

### Consent

Students were provided with information sheets and consent forms and following informed consent, the questionnaire. To maximise participation, those completing the questionnaire were offered entry into a prize draw to win an iPad. FE setting gatekeepers permission was sought from each participating institution including college managers, head teachers or deputy head teachers signing a memorandum of understanding to agree to take part in the research.

### Intervention

The intervention is described in accordance with the Template for Intervention Description and Replication (TIDieR) guidelines.^30^

### Name and brief description

**Figure 1:**
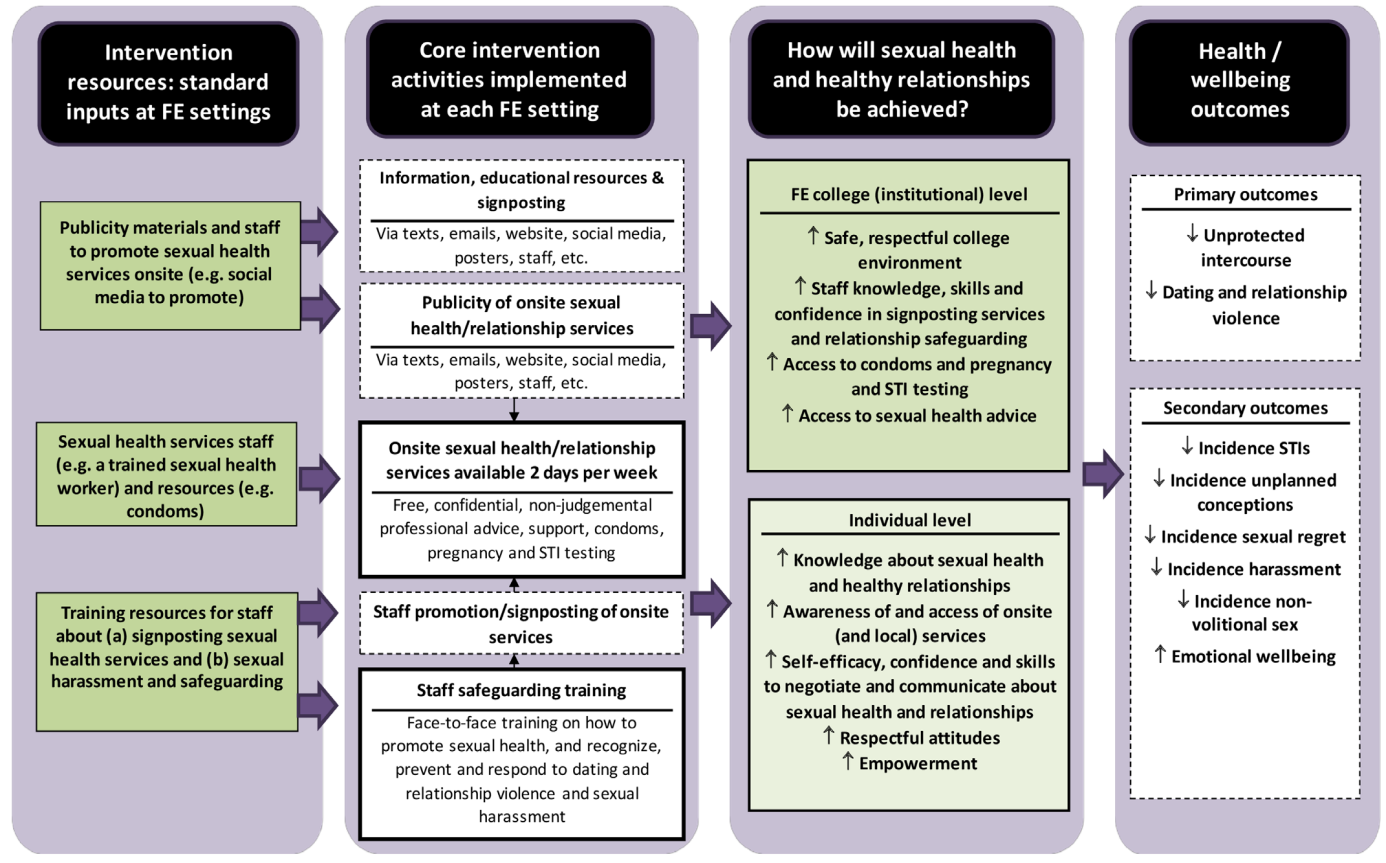
SaFE logic model. FE, further education; SaFE, Sexual health and healthy relationships for Further Education; STI, sexually transmitted infections.

Figure 1 shows the logic model for the SaFE intervention. SaFE aims to promote safer sex, prevent and manage DRV and sexual harassment in FE settings.

### Why, rationale of essential elements

The provision and promotion (via staff and publicity materials) of regular, free onsite sexual health services aimed to create an FE environment where positive sexual health and relationships were normalised. The aim was to increase students’ access to services; knowledge about sexual health, relationships and services and self-efficacy, confidence and skills about these topics. Social marketing principles were used to address the ‘4Ps’ selling consumers (students) a Product they want (sexual health and relationship services) in an accessible Place (their FE setting) at a low Price (free) with Promotion (via staff and publicity materials).^31^ In line with the social learning model,^32^ staff training aimed to provide skills to recognise, prevent and respond to DRV and sexual harassment, to challenge negative attitudes and social norms about DRV and sexual harassment in order to create safer and more respectful settings. Increasing staff and FE wide awareness and promoting appropriate behaviours attempted to shift norms about the acceptability and tolerance of these acts. Onsite services supported students’ skill development and behavioural control.

### What, a description of materials

The SaFE intervention has three components:

1. *Onsite access to sexual health and relationship services available for 2 hours on 2 days per week*. Services provided free, confidential access to non-judgemental, professional advice, support and signposting, condoms and pregnancy, chlamydia and gonorrhoea tests.^24^
2. *Publicity of onsite sexual health and relationship services*. Texts, emails, websites, social media, posters and events: (1) publicised onsite services and (2) gave information and educational resources about, and signposted to, local sexual health, relationship, DRV and sexual harassment services.
3. *FE staff training on how to promote sexual health, and recognise, prevent and respond to DRV and sexual harassment*. Online training was provided to FE staff on how to promote sexual health, recognise and respond to DRV and sexual harassment, and signpost students to (onsite) sexual health and relationship services. Training sought to help staff identify hotspots where DRV and harassment occur onsite. Training also included knowledge about how to manage sexual harassment at educational settings, and support or referral of victims or perpetrators to specialist services.

SaFE combined standardised inputs, processes and outputs but had flexibility to allow local adaptation to support universal adoption, institutional ownership and the implementation of multiple activities.^33^

### Who delivers the intervention?

FE staff training on DRV prevention and management was provided by a specialist practitioner with expertise in sexual health, domestic violence and safeguarding. Onsite sexual health services were provided by sexual health nurses. Onsite sexual health service publicity was coordinated by a nominated intervention champion, usually a member of the safeguarding or well-being team, in each FE setting.

### How, modes of delivery?

At least one (online) staff training session was provided to each intervention setting. Nurses attended FE settings to provide onsite sexual health services. Publicity materials were developed by the research team and provided to intervention sites. In some cases, FE settings developed their own posters and social media posts to publicise the services.

### Where, locations where intervention has occurred?

FE settings in south Wales and the west of England.

### Tailoring

FE staff training was interactive so staff could ask questions. Nurses provided onsite services where possible. In some cases, when they could not provide the service onsite (e.g., contraceptive pill/Pre-Exposure Prophylaxis), they arranged the appointment with another provider. Some FE settings modified or created their own materials publicising onsite sexual health services.

### Patient and public involvement

This study builds on 15 months of previously published work with over 2000 students and 200 staff from six FE settings, 12 sexual health staff and an advisory group of 16–21 year olds (ALPHA) to explore which components should be combined into an intervention.^26^ We discussed the findings, intervention and methods for this project with 30 stakeholders at a consultation event.

### Intervention funding

In Wales, funding was provided for intervention delivery by Health and Care Research Wales. In England, funding came from Public Health England.

### Outcome measures

The primary outcome of the pilot cRCT was whether progression to a phase III RCT is justified in terms of progression criteria (table 1). These criteria sought to address uncertainties in the intervention and cRCT design with thresholds set according to a traffic light system. All criteria being green would indicate that the uncertainties were addressed and the study should progress to a full-scale evaluation.

**Table 1:** Summary of results against the progression criteria

Progression criterion	Red	Amber	Green	Actual
At least 7 of the 8 FE settings are retained throughout the study.	<7		≥7	8
Percentage of students approached that complete a questionnaire at baseline and follow-up.	<50%	50–59%	≥60%	56.3%
The intervention is implemented with fidelity in at least 5 of 6 intervention settings.	<5		≥5	6
Percentage of sessions for onsite sexual health service a nurse attended a FE setting.	<50%	50–79%	≥80%	82.1%
Number of settings with onsite publicity of services.	<5	5	6	6
Number of settings where at least 5 members of staff attended training sessions.	<6		6	6 (137 staff trained across sites)
Intervention is acceptable to students, FE staff and public health commissioners.	Low	Medium	High	High

FEfurther education

We also examined the indicative primary outcomes of a future phase III trial. Unprotected intercourse at last intercourse was measured using validated questions from SHARE.^34^ Unprotected intercourse was defined as vaginal or anal (not oral) intercourse that involves no reliable method of STI and/or pregnancy prevention (i.e., reliable meaning STI prevention (e.g., condoms) and pregnancy prevention (e.g., condoms or other contraceptives if involving a girl/woman)). Self-reported experience of DRV victimisation in the last 12 months was measured using the 10-item short conflicts in adolescent dating relationships inventory (sCADRI).^35^

Informed by our logic model, the indicative self-reported secondary outcomes in a phase III RCT were: STI and pregnancy prevention methods used at last intercourse;^34 36^ use of emergency contraception at last intercourse;^34 36^ STI testing and diagnosis in the last 12 months;^34 36^ pregnancy and unintended pregnancy (initiation of pregnancy for boys/men) in the last 12 months;^34 36^ sexual harassment taking place at FE settings in the last 12 months using measures taken from the School Health Research Network survey^37^ and Hostile Hallways survey;^38^ non-volitional sex in the last 12 months using measures from the National Survey of Sexual Attitudes and Lifestyles (NATSAL);^39^ DRV perpetration in the last 12 months using the sCADRI as described above;^35^ EQ-5D-5L to measure health-related quality of life (reported elsewhere)^40^ and self-reported awareness of services, and help seeking for victims and perpetrators were assessed by existing measures.^41^

### Analysis

#### Statistical analysis

The primary analysis sought to determine whether the progression criteria to a full-scale phase III trial were met. The analyses were primarily descriptive, providing estimates of recruitment, response and retention rates. Recruitment, randomisation and retention of FE settings, as well as student recruitment, response, follow-up and consent to routine data linkage were summarised in a CONSORT flow diagram. We tabulated demographic characteristics of students within settings by study arm (intervention or control) and assessment time point (baseline or follow-up) using descriptive statistics: means and SDs (or medians and IQR, as appropriate) for continuous outcomes; and frequencies and percentages for discrete outcomes. Student recruitment, response, follow-up and consent to data linkage were tabulated by student-level socioeconomic disadvantage. We examined the rates of completion and discrimination (i.e., floor/ceiling effects) of indicative primary and secondary outcome measures for use in a full-scale phase III trial. We assessed the internal consistency of the scaled outcomes by reporting Cronbach’s alpha statistics at baseline and follow-up. Analysis was performed in Stata V.17.

#### Qualitative analysis

Qualitative data generated through semistructured interviews and focus groups were audio-recorded, transcribed and coded. Field-notes from observations and free-text entries in logbooks were coded using a similar system. Members of the research team (LC, RW-T and HY) analysed the data using inductive and deductive thematic analysis. To increase consistency, a coding scheme was developed by two researchers, using two interviews from each population group (e.g., FE students, FE staff, sexual health nurses) which were randomly chosen and based on the feasibility progression criteria. Each coding scheme included both a priori codes and in vivo codes. The coding scheme evolved during analysis, with the new codes discussed and confirmed by the team, before being applied to previously coded data. Disagreements between researchers were resolved through discussion. NVivo V.12 supported data analysis and storage. As part of the interpretative process of generating themes, visual maps were created. Overarching themes were presented to the wider study team who suggested further refinements of subthemes.

## Results

There were 1124 participants at baseline and 1139 at follow-up, with variation in student recruitment to take part in the survey across FE settings ranging from 41% to 100% at baseline and from 44% to 97% at follow-up. Of the recruited participants, 95.2% were 16–19 years of age, 54.4% were girls, 30.3% from an ethnic minority comprising Mixed or Multiple ethnicities, Pakistani, Indian, Bangladeshi, Chinese, African, Caribbean or Black and Arab; 96.5% lived with a parent or guardian, 74.1% were studying for an AS/A-level, 22% a Business and Technology Education Council (BTEC) qualification, and 13.5% the Welsh Baccalaureate, with multiple responses permitted. Overall, 89.3% were studying full time, and 46.2% were in part-time and 1.4% in full-time employment. There were few differences in participant characteristics across assessments or by arm (online supplemental etable 1). There were low levels of missing data for all demographic variables (i.e., <3%).

Table 1 summarises the results of the pilot cRCT against the progression criteria. Three of the four progression criteria were rated green and met. One criterion was rated amber: 56.3% of students approached completed a questionnaire whereas the green threshold was 60%.

1. At least seven of the eight FE settings are retained throughout the study.

All eight settings were retained throughout the study.

2. Percentage of students approached who complete a questionnaire at baseline and follow-up.

**Figure 2:**
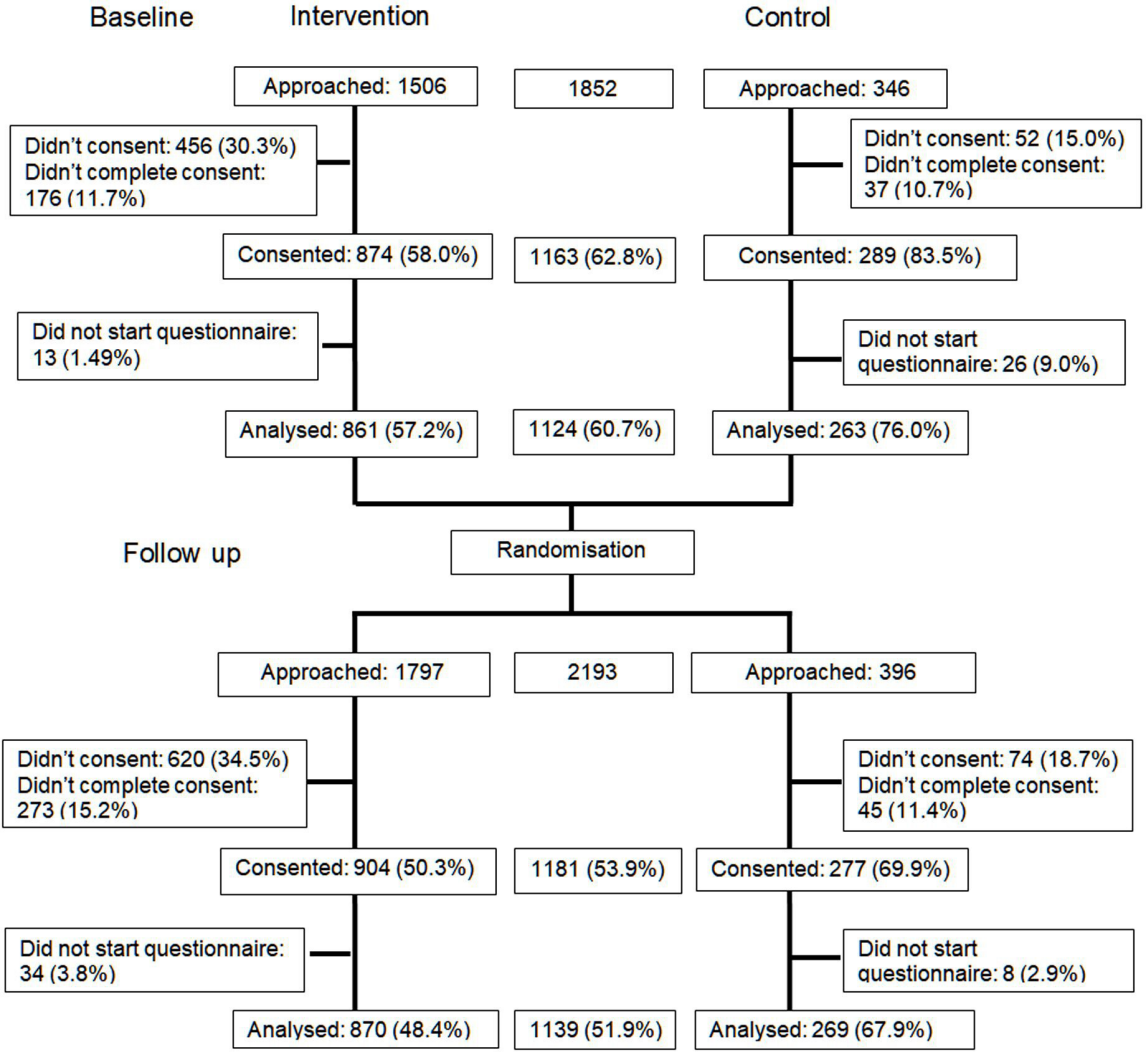
CONSORT flow diagram. CONSORT, Consolidated Standards of Reporting Trials.

Figure 2 shows the CONSORT flow diagram. The percentage of students approached who completed a questionnaire at baseline and follow-up was 56.3% (the ‘green’ threshold for this criterion was 60%). At baseline, of the 1852 students approached, 1124 (60.7%) completed the questionnaire. At follow-up, of the 2193 students approached, 1139 (51.9%) completed the questionnaire. The percentage of students approached who provided sufficient data so that it could be analysed was higher in the control than the intervention arm at baseline (76.0% vs 57.2.%) and follow-up (67.9% vs 48.4%). Of the baseline participants, 13.3% were resampled at follow-up.

3. The intervention is implemented with fidelity in at least five of the six intervention settings.

The intervention was delivered with fidelity in all six settings.

- *Percentage of sessions for onsite sexual health service a nurse attended an FE setting*. Of onsite sexual health services at FE settings, 82.1% were attended by a nurse. The number of sessions attended across sites ranged from 47.4% to 92.9%. The most frequent reason for students presenting at the service was to get condoms (38.0%), followed by advice (15.8%), pregnancy tests (15.8%) and STI tests (14.3%). Other reasons for attendance included STI treatment (4.8%), blood tests (4.8%), contraceptive implants (3.2%) and undisclosed safeguarding incidents unrelated to the intervention (3.2%).
- *Number of settings with onsite publicity of services*. Observations conducted by the research team found onsite publicity of sexual health services in all intervention settings. SaFE branded publicity materials were not used in one site due to their inconsistency with college branding; however, localised materials were developed detailing the same information. In all settings, publication materials were displayed in appropriate locations: main corridors, message boards, toilets, and common areas.
- *Number of settings where at least five members of staff attended training sessions*. At least five members of staff attended training in all six intervention sites. In total, 137 staff were trained across the six intervention sites. The 2 hour staff training was delivered using different methods due to restrictions on face-to-face contact during the COVID-19 pandemic and site preferences. Training sessions were provided through 2 hours face-to-face (one site), 2 hours online (three sites), two, 1 hour sessions over 1 week (two sites). The median number of staff attending each session was 19, minimum was six and maximum was 62.

4. The process evaluation indicates that the intervention is acceptable to students, FE staff and public health commissioners (measured by qualitative interview, routine monitoring data on attendance and survey data).

We identified four overarching themes, each with a set of related subthemes: (1) staff training, (2) reach and engagement, (3) acceptability, implementation, and potential improvements to safe intervention and (4) school/college provision of help for sexual health and DRV. Themes are organised by stakeholders to ensure all were represented.

### Students

Students liked that the sexual health provision was located onsite.

I feel like college will get you more help faster because you’re right, like you’re here aren’t you now. FE setting 2 (England), student focus group

But the location of the service within the setting was a barrier because they were concerned about being seen going into the service.

I think it’s good that it’s here, but I think it’s difficult because obviously people can see you in that room. FE setting 1 (England), student focus group

This feedback led the sexual health service to be moved to a more discreet, and preferred location in one intervention site, and the addition of a location in another intervention site (i.e., different rooms on different service days).

### Further education staff

Staff felt that training was beneficial as it increased their confidence in being able to respond to students if they want to discuss an issue with them.

I think it’s increased staff awareness when pupils are having conversations in class about relationships, etc. I think staff feel more confident that they can sort of challenge, you know, stereotypes, talk to pupils, you know, if, if you're in that position, perhaps you need to […] FE setting 2 (Wales), site lead interview

Yeah, definitely, yeah, I think it'd be really useful for anybody working, um, with students to have knowledge and training in those areas because that’s how they know, you know what to report and who to report it to, um, and how to safeguard students. FE setting 3 (England), staff

Most staff attending training were well-being staff or personal tutors who were most experienced dealing with DRV or harassment. Staff were therefore keen that in a future study, a wider variety of staff attended. Staff thought the onsite sexual health services would have had more attendance and reach if it had been delivered for longer and normalised as part of the well-being services within the sixth form or college.

More training slots to cover, to cover more staff… It’s only just starting to embed. FE setting 1 (Wales), site lead interview

But I think that potentially, the difference was just time. People just needed to get to know [Nurse] and the word get out. And you know, we often find them, that you can do some good marketing materials and put up posters and plasmas and whatever you want. But actually, it’s, it’s about that personal relationship. And one student going back and talking to their mate and saying, ‘ah, do you know what, I spoke to this really nice person’. FE setting 3 (England), site lead interview

Staff particularly valued having a sexual health nurse onsite to support students who had become pregnant.

And it’s been a godsend, particularly with the couple of pregnancies that we’ve had, that they happen to have come to us on the day that the sexual health clinic was there. FE setting 1 (Wales), site lead interview

In terms of implementing the training that the staff received, they reflected that the information on healthy relationships and pornography was really valuable. They were able to incorporate aspects into their teaching and disseminate the knowledge to their students.

Uh, the healthy relationships one absolutely. Um, I also took a lot of that information and put it into my, uh, lesson when I taught about healthy and unhealthy relationships. Um, the pornography one was also quite interesting as well, um, I didn't really create, like, a lesson on, uh, pornography, but I definitely did incorporate some of the, uh, issues into, uh, consent, uh, uh, my lesson consent, yeah, and of course consent was pretty useful as well. FE setting 3 (England), staff

With regard to the publicity of onsite sexual health services, staff thought that the use of posters was important in getting engagement from students, noting it was provided by a nurse and not FE staff.

I think obviously, the posters really highlight the service that was in place, and obviously, the specialist and provision provided by the school nurse. I think having that, rather than just teachers. … So, I think having that external person, makes the students feel a bit more comfortable in disclosing some of the issues potentially, just because they don’t want to necessarily disclose it to somebody they have to see every day. So, I think you should have an external person. FE setting 2 (England), site lead interview

### Nurses

Nurses also noted how finding an accessible but private room was important. However, too much privacy was also a barrier whereby students would not know if staff were available.

Yeah. And then when we, when they had a meeting and we and I mentioned about the room just not, not being really feasible, not in the right place.

Um, and then I think the following, they moved [Nurse1] and [Nurse1] had seen like four or five that day. Wales, nurse interview

Nurses felt COVID-19 pandemic restrictions had acted as a barrier to the service embedding and that it needed longer to embed.

So I think, do you know, I just think it wasn’t enough time, I think we came in at the wrong time for a start … So I think there was still the restrictions and everything so I think we’ve sort of had that barrier from the start, because COVID wasn’t over. Wales, nurse interview

Nurses felt that they needed more time to build trust with the students. The pandemic restrictions reduced their ability to see students and that students having a positive experience could lead to an increase in service use.

…like, and then I, you know, I seen one patient, then he brought his friend and, do you know, so it was, it, it, it, at the clinic I would just say it takes time to kick off. Wales, nurse interview

### Public health commissioners

The displacement of services from the NHS to FE settings was considered a positive move to enhance uptake for young people who may otherwise not be able to access services and provided a gateway into community services after leaving the setting. Improving FE staff skills on how to promote sexual health and prevent DRV was also seen as a positive feature of SaFE that could increase the reach of services.

I think it also matches priorities, really, um, the idea of developing the skills in that setting and making it relevant to, to that school and, and the children and young people, um, you know, in that, that school or college. So yeah, this, you know, it seems really promising and exciting. Wales, public health commissioner

### Routine monitoring exit checklist data

Exit checklists were optional for students leaving the nurses office after using the service. They were completed in four of the six intervention sites. A total of 18 checklists were completed across the four intervention sites; 100% of those who completed the checklist reported that they ‘got what they needed from their visit’ and 77.8% indicating that they would use the service again if they needed to.

### Outcome data

We found low rates of missing data for almost all variables with no major differences across arms. The highest rate of missing data on other variables was 14.8% in response to a question on whether participants had ever used a sexual health service in the intervention group at baseline.

The indicative primary outcomes were unprotected sex at last intercourse and DRV victimisation in the last 12 months using sCADRI.^35^ The prevalence of unprotected sex at last intercourse was 15.5% at baseline and 18.7% at the 12 month follow-up (online supplemental etable 2). There was an imbalance between the arms at baseline, with a higher prevalence in the intervention arm (17.5%) than in the control arm (7.1%). This difference was no longer apparent at 12 month follow-up (intervention 17.7% vs control 18.9%). There was evidence of floor effects for DRV victimisation in the last 12 months. The scale had a minimum score of 10.0. At baseline, the median score was 11.0 (IQR, 10.0–13.0) and at the 12 month follow-up of 10.0 (IQR, 10.0–13.0) (online supplemental etable 3). Cronbach alpha’s for DRV victimisation ranged from 0.92 to 0.97. Secondary outcomes are reported in online supplemental etables 4–9.

### Delivery costs

The estimated cost of delivering the SaFE intervention was approximately £38 363.09 per FE setting. This comprises £1610.40 (4.4%) for staff training and £36 752.69 (96.6%) for onsite sexual health and relationship services. We did not know the total number of students on site at any one time, so it is not possible to calculate an exact cost per student. Assuming a sixth form may have 150 students, this cost per setting would equate to £259.37 per student. A community college may have 2000 students which would reduce the cost per student to £19.45 per student.

### Intervention refinements

The process evaluation revealed several intervention refinements that could be made should the study proceed to a full-scale trial. Students felt it was important that onsite services were located somewhere that was discreet so people could not see them attend. In response to this feedback, the room used for onsite services was changed in two of the settings and nurses noted an increase in attendance. Nurses also noted the importance of room location but were concerned that the room was not visible enough and this was a barrier to attendance. In some FE settings, staff training was attended by well-being and safeguarding leads and a small number of other staff. Staff suggested that more, and varied staff needed to be trained and to do this by repeating training sessions. Recording the original training and making it freely available to all staff may help achieve greater reach among a more diverse range of FE staff. Other suggested changes included running the service for longer and without interruption so that students knew about the service and highlighting that it was run by nurses not FE staff.

## Discussion

The SaFE sexual health and DRV prevention intervention was delivered with fidelity in all six intervention FE settings. The process evaluation indicated that the intervention was acceptable to students, staff, the intervention delivery team and public health commissioners. All settings recruited were retained and 56.3% of students approached to participate agreed to do so, slightly below our target of 60%. All FE settings were retained in the study and there were low levels of missing data on outcomes. Three of the four progression criteria were met suggesting that there should be a follow-on full-scale cRCT of the SaFE intervention.

### Comparisons to existing studies

While there are some sexual health and healthy relationships interventions targeting 15–24 year olds, few have provided onsite sexual health services, and we are unaware of any rigorous evaluations of interventions which also provide training to FE staff on DRV prevention and management. Students wanted onsite services to be discreet and nurses noted the role of positive experiences by peers in the uptake of services. These findings replicate those from the ‘Test n Treat’ feasibility trial which offered free onsite rapid chlamydia/gonorrhoea tests at six technical colleges in London, UK.^42^ In both studies, students valued the accessibility of onsite services, but barriers included embarrassment and fear of stigma if they were seen accessing services. The paradoxical challenge of identifying rooms which are visible but discreet will need to be carefully considered when sexual health services are provided in FE setting.

### Feasibility of the pilot trial design

For the progression criteria relating to the acceptability of the cRCT design, all settings were recruited and retained at follow-up. We chose a repeat cross-sectional design as our previous work in FE settings found the high turnover of students, irregular days of student attendance and lack of accurate student enrolment data, made following the same students up 12 months later unlikely.^29^ In support of this assumption, only 13.3% of students recruited at baseline also completed a follow-up questionnaire. The one progression criterion that was not met related to student recruitment for the survey; 56.3% of those approached to take part consented to do so. The green threshold for this criterion was 60%. As a pilot, we had costed up to two follow-up visits to settings. Increasing the number of fieldworkers attending each setting and making more visits are likely to increase the number of participants beyond the additional 4% required to meet this criterion.

### Adaptations to the intervention

The process evaluation identified refinements that could be made to the SaFE intervention before it is tested further. These include carefully choosing the room for onsite sexual health services, running services for longer to allow them to embed and build student confidence and trust, and repeating staff training on DRV prevention and management. All are achievable refinements within the current intervention design.

### Strengths and limitations

We collected both quantitative and qualitative data to assess the acceptability of the SaFE intervention and trial methods. Focus group and interview data from FE staff, students and the intervention delivery team enabled an in-depth exploration of their experiences and views of the interventions. A further strength of the study was the contribution of key stakeholders to the delivery and implementation of the interventions.

We used a robust qualitative methodology for data collection and analysis. The students and staff who took part in the focus groups and interviews were, however, a self-selecting sample. Those who did not take part may have given different responses from those who chose to participate. For example, the students who volunteered may have been those who were more receptive to onsite sexual health services. However, students and intervention delivery staff were forthcoming when discussing what they did not like about the interventions. The estimate of the cost of the intervention was only based on six settings and is unlikely represent the true costs in a wider evaluation in a larger study. The costs per student differ depending on the number of students who attend FE settings. The lack of accurate data on the number of students attending colleges who were exposed to the intervention (particularly the 2 day onsite sexual health services) is a challenge to estimating exposure and costs per student, and the estimates of cost should be viewed in this light.

The timing of the research meant that the study had to be put on hold for a period during the COVID-19 pandemic and while restarting in January 2021, there was significant uncertainty about the conduct of education settings-based research, and the delivery of sexual health services. During this period, the study was impacted by staff and student sickness rates across both FE and service delivery teams, as well as impacts on student and staff attendance, and freedom to move throughout FE sites. Similarly, COVID-19 pandemic restrictions at FE settings meant that the intervention was not implemented for as long as planned (up to 23 weeks vs 39 weeks). In combination, this has adversely impacted the provision of the sexual health service, attendance at the service, as well as engagement of students with the completion of the baseline and follow-up surveys.

The study did not meet the ‘green’ threshold for one progression criterion relating student recruitment, whereby 60% of students approached to participate should have agreed to do so; in this case, 56.3% of students approached agreed to take part. COVID-19 and social/physical distancing may have impacted students’ engagement with the survey, and fewer students had returned to campus during the study; however, overall higher response rates were identified at baseline (60.7%; 1124/1852 students) compared with at 12 month follow-up (51.9%; 1139/2193 students). Before moving to a full-scale trial, the study team needs to work closely with young people to develop data collection methods and bespoke approaches to increase student engagement with the survey and increase the number of students consenting when approached to take part.

In line with previous research in FE settings,^29^ completion of the staff logbooks was very low across intervention sites. However, it is possible that in the case of the SaFE intervention, this was compounded by the additional administrative burdens placed on staff during the COVID-19 pandemic. More work needs to be done with FE staff to understand their competing demands and to explore how best to encourage and perhaps incentivise completion of logbooks in the event of student intervention relating to sexual health and dating and relationship and/or sexual harassment.

### Conclusion

SaFE is an acceptable FE-based intervention to promote sexual health and prevent DRV and sexual harassment, which can be delivered with high fidelity. The trial methods were acceptable with all settings recruited and retained. Some minor refinements in the intervention and trial methods would help to address the identified challenges to implementation and student recruitment prior to further testing in a larger trial.

## supplementary material

10.1136/bmjopen-2024-091355 online supplemental file 1

## Data Availability

Data are available upon reasonable request.
