# Supplementary material for: Sexual health and healthy relationships for Further Education (SaFE) in Wales and England: results from a pilot cluster randomised controlled trial
Source: BMJ Open. 2024 Dec 20;14(12):e091355. doi: 10.1136/bmjopen-2024-091355 (PMC11667339; doi:10.1136/bmjopen-2024-091355)
Supplement: online supplemental file 1 [file bmjopen-14-12-s001.docx]

**Supplementary materials**

1. eTable 1. Characteristics of the sample at baseline and 12 months follow-up by study group
2. eTable 2. Unprotected intercourse at last intercourse at baseline and 12 months follow-up by study group
3. eTable 3. Dating and relationship violence at baseline and 12 months follow-up by study group
4. eTable 4. Secondary outcome of STI and pregnancy prevention methods
5. eTable 5. Secondary outcome of sexual health checks, sexual health services use, and sexually transmitted infections
6. eTable 6. Secondary outcome of self-reported pregnancy and unintended pregnancy (initiation of pregnancy for boys/men)
7. eTable 7. Secondary outcome of sexual harassment taking place at further education settings
8. eTable 8. Secondary outcome of relationship with partner (sCADRI)
9. eTable 9. Secondary outcome assessed in intervention group only: awareness and potential use of the service

**eTable 1: Characteristics of the sample at baseline and 12 months follow-up by study group**

|  | **Baseline** | | | | | | | **12 Months Follow-up** | | | | | |  |
| --- | --- | --- | --- | --- | --- | --- | --- | --- | --- | --- | --- | --- | --- | --- |
|  | **Overall (N=1124)** | | **Control (N=263)** | | **Intervention (N=861)** | | | **Overall (N=1139)** | | **Control (N=269)** | | **Intervention (N=870)** | | |
|  | **n** | **%** | **n** | **%** | **n** | | **%** | **n** | **%** | **n** | **%** | **n** | **%** | |
| **Age in years:** | | | | | | | |  |  |  |  |  |  | |
| 16 | 451 | 40.1 | 108 | 41.1 | 343 | | 39.8 | 507 | 44.5 | 116 | 43.1 | 391 | 44.9 | |
| 17 | 480 | 42.7 | 114 | 43.3 | 366 | | 42.5 | 480 | 42.1 | 126 | 46.8 | 354 | 40.7 | |
| 18 | 128 | 11.4 | 36 | 13.7 | 92 | | 10.7 | 84 | 7.4 | 27 | 10.0 | 57 | 6.6 | |
| 19+ | 65 | 5.8 | 5 | 1.9 | 60 | | 7.0 | 68 | 6.0 | 0 | 0.0 | 68 | 7.8 | |
| Missing | 0 | 0.0 | 0 | 0.0 | 0 | | 0.0 | 0 | 0.0 | 0 | 0.0 | 0 | 0.0 | |
| **Gender ^a^:** |  |  |  |  |  | |  |  |  |  |  |  |  | |
| Boy | 479 | 42.6 | 120 | 45.6 | 359 | | 41.7 | 469 | 41.2 | 104 | 38.7 | 365 | 42.0 | |
| Girl | 609 | 54.2 | 136 | 51.6 | 473 | | 54.9 | 614 | 53.9 | 157 | 58.3 | 457 | 52.5 | |
| Gender minority | 33 | 2.9 | 5 | 2.0 | 28 | | 3.3 | 50 | 4.3 | 8 | 3.0 | 42 | 4.9 | |
| Prefer not to say | 3 | 0.3 | 2 | 0.8 | 1 | | 0.1 | 5 | 0.4 | 0 | 0.0 | 5 | 0.5 | |
| Missing | 0 | 0.0 | 0 | 0.0 | 0 | | 0.0 | 1 | 0.1 | 0 | 0.0 | 1 | 0.1 | |
| **Ethnicity ^b^:** |  |  |  |  |  | |  |  |  |  |  |  |  | |
| White British | 839 | 74.6 | 137 | 52.1 | 702 | | 81.6 | 826 | 72.5 | 143 | 53.2 | 683 | 78.5 | |
| Ethnic minority | 278 | 24.8 | 121 | 46.0 | 157 | | 18.2 | 302 | 26.5 | 123 | 45.7 | 179 | 20.6 | |
| I do not want to answer | 6 | 0.5 | 5 | 1.9 | 1 | | 0.1 | 10 | 0.9 | 3 | 1.1 | 7 | 0.8 | |
| Missing | 1 | 0.1 | 0 | 0.0 | 1 | | 0.1 | 1 | 0.1 | 0 | 0.0 | 1 | 0.1 | |
| **Live with ^c^:** |  |  |  |  |  | |  |  |  |  |  |  |  | |
| Parent/ Guardian | 1079 | 96.0 | 257 | 98.1 | 822 | | 95.7 | 1092 | 95.9 | 265 | 98.5 | 827 | 95.1 | |
| Alone | 8 | 0.7 | 2 | 0.8 | 6 | | 0.7 | 13 | 1.1 | 0 | 0.0 | 13 | 1.5 | |
| Friends / Housemates | 4 | 0.4 | 2 | 0.8 | 2 | | 0.2 | 8 | 0.7 | 0 | 0.0 | 8 | 0.9 | |
| Boyfriend / Girlfriend | 18 | 1.6 | 1 | 0.4 | 17 | | 2.1 | 11 | 1.0 | 0 | 0.0 | 11 | 1.3 | |
| Foster / Local authority care | 3 | 0.3 | 2 | 0.8 | 1 | | 0.1 | 7 | 0.6 | 1 | 0.4 | 6 | 0.7 | |
| Grandparents | 16 | 1.4 | 2 | 0.8 | 14 | | 1.7 | 18 | 1.6 | 3 | 1.1 | 15 | 1.7 | |
| Other | 17 | 1.5 | 0 | 0.0 | 17 | | 2.1 | 14 | 1.2 | 5 | 1.9 | 9 | 1.0 | |
| Missing | 1 | 0.1 | 0 | 0.0 | 1 | | 0.1 | 3 | 0.3 | 1 | 0.4 | 2 | 0.2 | |
| **Live with adult who is in paid employment:** | | | | | |  | |  |  |  |  |  |  | |
| Yes | 967 | 86.0 | 223 | 84.8 | 744 | | 86.4 | 991 | 87.0 | 238 | 88.5 | 753 | 86.6 | |
| No | 95 | 8.5 | 26 | 9.9 | 69 | | 8.0 | 77 | 6.8 | 18 | 6.7 | 59 | 6.8 | |
| Not sure | 47 | 4.2 | 9 | 3.4 | 38 | | 4.4 | 43 | 3.8 | 10 | 3.7 | 33 | 3.8 | |
| Missing | 15 | 1.3 | 5 | 1.9 | 10 | | 1.2 | 28 | 2.5 | 3 | 1.1 | 25 | 2.9 | |
| **Qualification currently studying for ^c^:** | | | | | |  | |  |  |  |  |  |  | |
| Access level course | 87 | 7.7 | 9 | 3.7 | 78 | | 9.1 | 93 | 8.2 | 21 | 7.8 | 72 | 8.3 | |
| Apprenticeship | 10 | 0.9 | 0 | 0.0 | 10 | | 1.2 | 21 | 1.8 | 1 | 0.4 | 20 | 2.3 | |
| AS/A-Level | 841 | 74.8 | 214 | 82.9 | 627 | | 72.8 | 804 | 70.6 | 200 | 74.3 | 604 | 69.4 | |
| BTEC | 247 | 22.0 | 122 | 48.8 | 125 | | 14.5 | 246 | 21.6 | 118 | 43.9 | 128 | 14.7 | |
| Essential Skills | 6 | 0.5 | 0 | 0.0 | 6 | | 0.7 | 8 | 0.7 | 1 | 0.4 | 7 | 0.8 | |
| Foundation Degree | 7 | 0.6 | 1 | 0.4 | 6 | | 0.7 | 4 | 0.4 | 0 | 0.0 | 4 | 0.5 | |
| GCSE | 43 | 3.8 | 19 | 7.7 | 24 | | 2.8 | 57 | 5.0 | 10 | 3.7 | 47 | 5.4 | |
| HNC (Higher National Certificate) | 6 | 0.5 | 3 | 1.2 | 3 | | 0.3 | 7 | 0.6 | 2 | 0.7 | 5 | 0.6 | |
| HND (Higher National Diploma) | 9 | 0.8 | 0 | 0.0 | 9 | | 1.0 | 17 | 1.5 | 0 | 0.0 | 17 | 2.0 | |
| Welsh Baccalaureate | 171 | 15.2 | 12 | 4.9 | 159 | | 18.5 | 184 | 16.2 | 20 | 7.4 | 164 | 18.9 | |
| Other (Cambridge Technical, Exchange student, Vocational) | 18 | 1.6 | 2 | 0.8 | 16 | | 1.9 | 54 | 4.7 | 6 | 2.2 | 48 | 5.5 | |
| Missing | 6 | 0.5 | 1 | 0.4 | 5 | | 0.6 | 8 | 0.7 | 2 | 0.7 | 6 | 0.7 | |
| **Studying:** | | | | | |  | |  |  |  |  |  |  | |
| Full-time | 1032 | 91.8 | 231 | 87.8 | 801 | | 93.0 | 1015 | 89.1 | 224 | 83.3 | 791 | 90.9 | |
| Part-time | 85 | 7.6 | 27 | 10.3 | 58 | | 6.7 | 119 | 10.4 | 44 | 16.4 | 75 | 8.6 | |
| Missing | 7 | 0.6 | 5 | 1.9 | 2 | | 0.2 | 5 | 0.4 | 1 | 0.4 | 4 | 0.5 | |
| **In paid employment:** | | | | | |  | |  |  |  |  |  |  | |
| Yes, full-time (35 or more hours per week) | 13 | 1.2 | 1 | 0.4 | 12 | | 1.4 | 23 | 2.0 | 3 | 1.1 | 20 | 2.3 | |
| Yes, part-time (less than 35 hours per week) | 532 | 47.3 | 95 | 36.1 | 437 | | 50.8 | 561 | 49.3 | 116 | 43.1 | 445 | 51.1 | |
| No | 573 | 51.0 | 165 | 62.7 | 408 | | 47.4 | 551 | 48.4 | 150 | 55.8 | 401 | 46.1 | |
| Missing | 6 | 0.5 | 2 | 0.8 | 4 | | 0.5 | 4 | 0.4 | 0 | 0.0 | 4 | 0.5 | |

^a^ Gender minority comprises trans boy, trans girl, non-binary (neither boy or girl), unsure / questioning, or other. ^b^ Ethnic minority comprises white other, mixed or multiple ethnic groups, Pakistani, Indian, Bangladeshi, Chinese, African, Caribbean or Black, Arab, other. ^c^ The categories of this variable are not mutually exclusive (i.e., multiple options were allowed to be selected).

**eTable 2. Primary outcome of having last time sex without any contraception and without condoms**

|  | **Baseline** | | | | | | **12 Months Follow-up** | | | | | |
| --- | --- | --- | --- | --- | --- | --- | --- | --- | --- | --- | --- | --- |
|  | **Overall (N=325^a^)** | | **Control (N=56)** | | **Intervention (N=269)** | | **Overall**  **(N=315 ^a^)** | | **Control (N=51)** | | **Intervention (N=264)** | |
|  | **n** | **%** | **n** | **%** | **n** | **%** | **n** | **%** | **n** | **%** | **n** | **%** |
| **Having last time sex without contraception ^b^** | 51 | 15.7 | 4 | 7.1 | 47 | 17.5 | 59 | 18.7 | 9 | 17.7 | 50 | 18.9 |
| **Missing** | 10 | 3.1 | 0 | 0.0 | 10 | 3.7 | 3 | 1.0 | 0 | 0.0 | 3 | 1.1 |
| **Having last time sex without condoms ^c^** | 165 | 50.9 | 25 | 44.6 | 140 | 52.2 | 165 | 52.4 | 29 | 56.9 | 136 | 51.5 |
| **Missing** | 1 | 0.3 | 0 | 0.0 | 1 | 0.4 | 3 | 1.0 | 0 | 0.0 | 3 | 1.1 |

**^a^** In this table, the analysis is based on the sample of those participants who reported to “have ever had vaginal and/or anal sex” (N=325 baseline sample, N=315 follow-up sample, see eTable 4).

^b^ A participant is considered to have had last time sex without contraception if they responded, “No method used” to the question “The last time you had sex, did you or your partner use any form of contraception?” and did not report any contraceptive use in the subsequent options.

^c^ A participant is considered to have had last time sex without condoms if the option “Condoms used” as a response to the question “The last time you had sex, did you or your partner use any form of contraception?” was selected by the participant.

**eTable 3. Primary outcome of dating and relationship violence**

|  | **Baseline** | | | | | | | **12 Months Follow-up** | | | | | | | | | | | | |
| --- | --- | --- | --- | --- | --- | --- | --- | --- | --- | --- | --- | --- | --- | --- | --- | --- | --- | --- | --- | --- |
|  | **Overall (N=643^a^)** | | **Control (N=125)** | | | **Intervention (N=518)** | | **Overall (N=674^a^)** | | | | **Control (N=125)** | | | | **Intervention (N=549)** | | | | |
|  | n | % | n | % | n | | % | n | % | n | | | % | n | | | % |  |  |  |
| **Participant being a victim of partner’s violence ^b^:** | | | | | | | |  |  | |  |  | | |  |  | | |  |  |
| Sexual abuse | 79 | 12.2 | 15 | 12.0 | 64 | | 12.4 | 66 | 9.8 | 7 | | | 5.6 | 59 | | | 10.8 |  |  |  |
| Missing | 5 | 0.9 | 1 | 0.8 | 4 | | 0.8 | 2 | 0.3 | 1 | | | 0.8 | 1 | | | 0.2 |  |  |  |
| Physical abuse | 63 | 9.8 | 13 | 10.4 | 50 | | 9.7 | 69 | 10.2 | 16 | | | 12.8 | 53 | | | 9.7 |  |  |  |
| Missing | 4 | 0.6 | 1 | 0.8 | 3 | | 0.6 | 2 | 0.3 | 1 | | | 0.8 | 1 | | | 0.2 |  |  |  |
| Threatening behaviour | 57 | 8.9 | 11 | 8.8 | 46 | | 8.9 | 54 | 8.0 | 9 | | | 7.2 | 45 | | | 8.2 |  |  |  |
| Missing | 4 | 0.6 | 1 | 0.8 | 3 | | 0.6 | 2 | 0.3 | 1 | | | 0.8 | 1 | | | 0.2 |  |  |  |
| Relational abuse | 107 | 16.7 | 16 | 12.8 | 91 | | 17.6 | 133 | 19.7 | 26 | | | 20.8 | 107 | | | 19.5 |  |  |  |
| Missing | 3 | 0.5 | 1 | 0.8 | 2 | | 0.4 | 2 | 0.3 | 1 | | | 0.8 | 1 | | | 0.2 |  |  |  |
| Verbal and emotional abuse | 258 | 40.1 | 47 | 37.6 | 211 | | 40.7 | 268 | 39.8 | 50 | | | 40.0 | 218 | | | 39.7 |  |  |  |
| Missing | 3 | 0.5 | 1 | 0.8 | 2 | | 0.4 | 2 | 0.3 | 1 | | | 0.8 | 1 | | | 0.2 |  |  |  |
| Overall DRV | 295 | 45.9 | 58 | 46.8 | 237 | | 45.8 | 310 | 46.0 | 63 | | | 50.4 | 247 | | | 45.0 |  |  |  |
| Missing | 3 | 0.5 | 1 | 0.8 | 2 | | 0.4 | 2 | 0.3 | 1 | | | 0.8 | 1 | | | 0.2 |  |  |  |
| Total scale score **^c^**:  Median (IQR)  Mean (SD)  Min-Max  Missing | 11  13.9    9 | (10-13)  (8.6)  10-50  (1.4) | 11  13.8    4 | (10-13)  (8.5)  10-50  (3.2) | 11  13.9    5 | | (10-14)  (8.6)  10-50  (1.0) | 10  13.4    5 | (10-13)  (7.4)  10-50   (0.7) | 11  12.7    2 | | | (10-13)  (5.7)  10-50 (1.6) | 10  13.6    3 | | | (10-14)  (7.7)  10-50  (0.5) |  |  |  |
| **Participant being a perpetrator to their partner^b^:** | | | | | | | |  |  | |  |  | | |  |  | | |  |  |
| Sexual abuse | 7 | 1.1 | 3 | 2.4 | 4 | | 0.8 | 5 | 0.7 | 1 | | | 0.8 | 4 | | | 0.7 |  |  |  |
| Missing | 11 | 1.7 | 2 | 1.6 | 9 | | 1.7 | 5 | 0.7 | 1 | | | 0.8 | 4 | | | 0.7 |  |  |  |
| Physical abuse | 23 | 3.6 | 7 | 5.6 | 16 | | 3.1 | 24 | 3.6 | 4 | | | 3.2 | 20 | | | 3.6 |  |  |  |
| Missing | 6 | 0.9 | 1 | 0.8 | 5 | | 1.0 | 4 | 0.6 | 1 | | | 0.8 | 3 | | | 0.6 |  |  |  |
| Threatening behaviour | 12 | 1.9 | 2 | 1.6 | 10 | | 1.9 | 14 | 2.1 | 3 | | | 2.4 | 11 | | | 2.0 |  |  |  |
| Missing | 8 | 1.2 | 1 | 0.8 | 7 | | 1.4 | 4 | 0.6 | 1 | | | 0.8 | 3 | | | 0.6 |  |  |  |
| Relational abuse | 26 | 4.0 | 5 | 4.0 | 21 | | 4.1 | 38 | 5.6 | 8 | | | 6.4 | 30 | | | 5.5 |  |  |  |
| Missing | 5 | 0.8 | 1 | 0.8 | 4 | | 0.8 | 4 | 0.6 | 1 | | | 0.8 | 3 | | | 0.6 |  |  |  |
| Verbal and emotional abuse | 183 | 28.5 | 34 | 27.2 | 149 | | 28.8 | 195 | 28.9 | 41 | | | 32.8 | 154 | | | 28.1 |  |  |  |
| Missing | 4 | 0.6 | 1 | 0.8 | 3 | | 0.6 | 4 | 0.6 | 1 | | | 0.8 | 3 | | | 0.6 |  |  |  |
| Overall DRV | 203 | 31.6 | 38 | 30.4 | 165 | | 31.9 | 213 | 31.6 | 44 | | | 35.2 | 169 | | | 30.8 |  |  |  |
| Missing | 4 | 0.6 | 1 | 0.8 | 3 | | 0.6 | 4 | 0.6 | 1 | | | 0.8 | 3 | | | 0.6 |  |  |  |
| Total scale score **^c^**: Median (IQR)  Mean (SD)  Min-Max  Missing | 10  12.1    20 | (10-11)  (7.4)  10-50  (3.1) | 10  12.3    4 | (10-11)  (7.8)  10-50  (3.2) | 10  12.1    16 | | (10-11)  (7.4)  10-50  (3.1) | 10  11.6    17 | (10-11)  (6.2)  10-50  (2.5) | 10  11.0    2 | | | (10-11)  (3.7)  10-50  (1.6) | 10  11.8    15 | | | (10-11)  (6.6)  10-50  (2.7) |  |  |  |

**^a^** In this table, the analysis is based on the sample of those participants who reported to have ever dated or been in a relationship (i.e., currently or in the past, see eTable 4), baseline N=643 (314+329) and follow-up N=674 (309+365).

**^b^** Frequencies and percentages are based on the binary versions of the original responses (original rated items) coded as [2 to 4=1 (Yes), 1 & 5=0 (No)].  Hence, the overall DRV and the sub-domains are defined as at least one item of the sCADRI being positive. For example, the item of “My partner spoke to me in a hostile/mean tone of voice” was considered as positive for a participant if the response was either “2. rarely”, “3. sometimes”, or “4. often”, which was then coded as 1 (Yes).

**^c^** Total score is computed by adding responses of all sCADRI items. The score is missing for a participant if their response to at least one item in the scale is missing.

**eTable 4. Secondary outcomes of STI and pregnancy prevention methods**

|  | **Baseline** | | | | | | **12 Months Follow-up** | | | | | |  |
| --- | --- | --- | --- | --- | --- | --- | --- | --- | --- | --- | --- | --- | --- |
|  | **Overall (N=325 ^a^)** | | **Control (N=56)** | | **Intervention (N=269)** | | **Overall (N=315 ^a^)** | | **Control (N=51)** | | **Intervention (N=264)** | |  |
|  | **n** | **%** | **N** | **%** | **N** | **%** | **n** | **%** | **n** | **%** | **n** | **%** |  |
| **The last time you had sex, did you or your partner use any form of contraception?** | | | | | | |  |  |  |  |  |  | |
| No method used | 55 | 16.9 | 6 | 10.7 | 49 | 18.2 | 65 | 20.6 | 10 | 19.6 | 55 | 20.8 |  |
| **The last time you had sex, did you or your partner use ^b^:** | | | | | | |  |  |  |  |  |  | |
| Condoms | 159 | 48.9 | 31 | 55.4 | 128 | 47.6 | 147 | 46.7 | 22 | 43.1 | 125 | 47.4 |  |
| Oral contraceptive pill | 103 | 31.7 | 25 | 44.6 | 78 | 29.0 | 92 | 29.2 | 22 | 43.1 | 70 | 26.5 |  |
| Intrauterine device (IUD, copper coil, hormonal IUS) | 11 | 3.4 | 2 | 3.6 | 9 | 3.4 | 11 | 3.5 | 2 | 3.9 | 9 | 3.4 |  |
| Vaginal ring (e.g. NuvaRing). | 0 | 0.0 | 0 | 0.0 | 0 | 0.0 | 2 | 0.6 | 1 | 2.0 | 1 | 0.4 |  |
| Contraceptive patch (e.g. Evra) | 1 | 0.3 | 0 | 0.0 | 1 | 0.4 | 2 | 0.6 | 1 | 2.0 | 1 | 0.4 |  |
| Injections | 8 | 2.5 | 1 | 1.8 | 7 | 2.6 | 8 | 2.5 | 1 | 2.0 | 7 | 2.7 |  |
| Implants | 32 | 9.8 | 4 | 7.1 | 28 | 10.4 | 27 | 8.6 | 7 | 13.7 | 20 | 7.6 |  |
| Emergency contraceptive pill/morning after pill | 3 | 0.9 | 0 | 0.0 | 3 | 1.1 | 6 | 1.9 | 2 | 3.9 | 4 | 1.5 |  |
| Safe period/calendar method/rhythm method | 6 | 1.8 | 0 | 0.0 | 6 | 2.2 | 6 | 1.9 | 2 | 3.9 | 4 | 1.5 |  |
| Withdrawal (partner not ejaculating in vagina) | 23 | 7.1 | 6 | 10.7 | 17 | 6.3 | 22 | 7.0 | 3 | 5.9 | 19 | 7.2 |  |
| Spermicides (foams/gels/sprays/pessaries) | 0 | 0.0 | 0 | 0.0 | 0 | 0.0 | 1 | 0.3 | 1 | 2.0 | 0 | 0.0 |  |
| Other contraception | 4 | 1.2 | 0 | 0.0 | 4 | 1.5 | 4 | 1.3 | 1 | 2.0 | 3 | 1.1 |  |
| Prefer not to say | 2 | 0.6 | 0 | 0.0 | 2 | 0.7 | 5 | 1.6 | 2 | 3.9 | 3 | 1.1 |  |
| Does not apply – currently pregnant | 0 | 0.0 | 0 | 0.0 | 0 | 0.0 | 2 | 0.6 | 1 | 2.0 | 1 | 0.4 |  |
| Does not apply – trying to get pregnant | 0 | 0.0 | 0 | 0.0 | 0 | 0.0 | 1 | 0.3 | 1 | 2.0 | 0 | 0.0 |  |
| Does not apply – cannot get pregnant | 4 | 1.2 | 1 | 1.8 | 3 | 1.1 | 4 | 1.3 | 1 | 2.0 | 3 | 1.1 |  |
| Missing | 1 | 0.3 | 0 | 0.0 | 1 | 0.4 | 3 | 1.0 | 0 | 0.0 | 3 | 1.1 |  |

**^a^** The categories of this variable are not mutually exclusive (i.e., multiple options were allowed to be selected if applicable).

**^b^** In this table, the analysis is based on the sample of those participants who reported to “have ever had vaginal and/or anal sex” (N=325 baseline sample, N=315 follow-up sample).

**eTable 5. Secondary outcome of sexual health checks, sexual health services use, and sexually transmitted infections**

|  | **Baseline** | | | | | | | **12 Months Follow-up** | | | | | | | | | | | |
| --- | --- | --- | --- | --- | --- | --- | --- | --- | --- | --- | --- | --- | --- | --- | --- | --- | --- | --- | --- |
|  | **Overall (N=1124)** | | **Control (N=263)** | | **Intervention (N=861)** | | | **Overall (N=1139)** | | | | **Control (N=269)** | | | | **Intervention (N=870)** | | | |
|  | **n** | **%** | **n** | **%** | **n** | **%** | | **n** | | **%** | | **n** | | **%** | | **N** | | **%** | |
| **Have you ever used a sexual health service:** | | | | | | |  | |  | |  | |  | |  | |  | |  |
| Yes | 167 | 14.9 | 30 | 11.4 | 137 | 15.9 | | 159 | | 14.0 | | 30 | | 11.2 | | 129 | | 14.8 | |
| No | 798 | 71.0 | 201 | 76.4 | 597 | 69.3 | | 830 | | 72.9 | | 215 | | 79.9 | | 615 | | 70.7 | |
| Missing | 159 | 14.1 | 32 | 12.2 | 127 | 14.8 | | 150 | | 13.2 | | 24 | | 8.9 | | 126 | | 14.5 | |
| **Have you ever been tested for chlamydia:** | | | | | | |  | |  | |  | |  | |  | |  | |  |
| Yes | 50 | 4.4 | 8 | 3.0 | 42 | 4.9 | | 40 | | 3.5 | | 5 | | 1.9 | | 35 | | 4.0 | |
| No | 920 | 81.9 | 230 | 87.5 | 690 | 80.1 | | 936 | | 82.2 | | 239 | | 88.8 | | 697 | | 80.1 | |
| Don’t know | 10 | 0.9 | 2 | 0.8 | 8 | 0.9 | | 14 | | 1.2 | | 3 | | 1.1 | | 11 | | 1.3 | |
| Prefer not to say | 27 | 2.4 | 23 | 8.7 | 4 | 0.5 | | 3 | | 0.3 | | 1 | | 0.4 | | 2 | | 0.2 | |
| Missing | 117 | 10.4 | 0.0 | 0.0 | 117 | 13.6 | | 146 | | 12.8 | | 21 | | 7.8 | | 125 | | 14.4 | |
| **Have you ever been tested for HIV:** | | | | | | |  | |  | |  | |  | |  | |  | |  |
| Yes | 29 | 2.6 | 2 | 0.8 | 27 | 3.1 | | 29 | | 2.5 | | 2 | | 0.7 | | 27 | | 3.1 | |
| No | 939 | 83.5 | 235 | 89.4 | 704 | 81.8 | | 939 | | 82.4 | | 239 | | 88.8 | | 700 | | 80.5 | |
| Don’t know | 11 | 1.0 | 3 | 1.1 | 8 | 0.9 | | 19 | | 1.7 | | 4 | | 1.5 | | 15 | | 1.7 | |
| Prefer not to say | 2 | 0.2 | 0 | 0.0 | 2 | 0.2 | | 2 | | 0.2 | | 0 | | 0.0 | | 2 | | 0.2 | |
| Missing | 143 | 12.7 | 23 | 8.7 | 120 | 13.9 | | 150 | | 13.2 | | 24 | | 8.9 | | 126 | | 14.5 | |
| **Have you ever been tested for other sexually transmitted infection:** | | | | | | |  | |  | |  | |  | |  | |  | |  |
| Yes | 43 | 3.8 | 7 | 2.7 | 36 | 4.2 | | 35 | | 3.1 | | 2 | | 0.7 | | 33 | | 3.8 | |
| No | 931 | 82.8 | 231 | 87.8 | 700 | 81.3 | | 940 | | 82.5 | | 242 | | 90.0 | | 698 | | 80.2 | |
| Don’t know | 5 | 0.4 | 2 | 0.8 | 3 | 0.3 | | 12 | | 1.1 | | 1 | | 0.4 | | 11 | | 1.3 | |
| Prefer not to say | 1 | 0.1 | 0 | 0.0 | 1 | 0.1 | | 2 | | 0.2 | | 0 | | 0.0 | | 2 | | 0.2 | |
| Missing | 144 | 12.8 | 23 | 8.7 | 121 | 14.1 | | 150 | | 13.2 | | 24 | | 8.9 | | 126 | | 14.5 | |
| **Have you ever been told by a healthcare professional or service that you had any of chlamydia, syphilis, genital herpes, gonorrhoea, HIV, any other:** | | | | | | |  | |  | |  | |  | |  | |  | |  |
| Yes | 14 | 1.2 | 2 | 0.8 | 12 | 1.4 | | 12 | | 1.1 | | 0 | | 0.0 | | 12 | | 1.4 | |
| No | 1004 | 89.3 | 242 | 92.0 | 762 | 88.5 | | 1023 | | 89.8 | | 248 | | 92.2 | | 775 | | 89.1 | |
| Missing | 106 | 9.4 | 19 | 7.2 | 87 | 10.1 | | 104 | | 9.1 | | 21 | | 7.8 | | 83 | | 9.5 | |
| **Please select all that apply ^a^:** | | | | | | |  | |  | |  | |  | |  | |  | |  |
| Chlamydia | 9 | 64.3 | 1 | 50.0 | 8 | 66.7 | | 6 | | 50.0 | | 0 0.0 | |  | | 6 | | 50.0 | |
| Syphilis | 0 | 0.0 | 0 | 0.0 | 0 | 0.0 | | 2 | | 16.7 | | 0 0.0 | |  | | 2 | | 16.7 | |
| Genital herpes | 3 | 21.4 | 1 | 50.0 | 2 | 16.7 | | 3 | | 25.0 | | 0 0.0 | |  | | 3 | | 25.0 | |
| Gonorrhoea | 0 | 0.0 | 0 | 0.0 | 0 | 0.0 | | 2 | | 16.7 | | 0 0.0 | |  | | 2 | | 16.7 | |
| HIV | 0 | 0.0 | 0 | 0.0 | 0 | 0.0 | | 2 | | 16.7 | | 0 0.0 | |  | | 2 | | 16.7 | |
| Yes, but can’t remember which one | 1 | 7.1 | 0 | 0.0 | 1 | 8.3 | | 2 | | 16.7 | | 0 0.0 | |  | | 2 | | 16.7 | |
| Other (genital warts, HPV, the clap) | 2 | 14.3 | 0 | 0.0 | 2 | 16.7 | | 5 | | 41.7 | | 0 0.0 | |  | | 5 | | 41.7 | |

**^a^** The categories of this variable are not mutually exclusive (i.e., multiple options were allowed to be selected if applicable).

**eTable 6. Secondary outcome of self-reported pregnancy and unintended pregnancy (initiation of pregnancy for boys/men)**

|  | **Baseline** | | | | | | | **12 Months Follow-up** | | | | | | | | | | |  |
| --- | --- | --- | --- | --- | --- | --- | --- | --- | --- | --- | --- | --- | --- | --- | --- | --- | --- | --- | --- |
|  | **Overall (N=325 ^a^)** | | **Control (N=56)** | | **Intervention (N=269)** | | | **Overall (N=315 ^a^)** | | | | **Control (N=51)** | | | | **Intervention (N=264)** | | |  |
|  | **n** | **%** | **n** | **%** | **n** | **%** | | **n** | | **%** | | **n** | | **%** | | **n** | | **%** |  |
| **Have you ever been pregnant or got a sexual partner pregnant?** | | | | | | |  | |  | |  | |  | |  | |  | |  |
| Yes | 32 | 9.9 | 1 | 1.8 | 31 | 11.5 | | 21 | | 6.7 | | 1 | | 2.0 | | 20 | | 7.6 |  |
| No | 272 | 83.7 | 53 | 94.6 | 219 | 81.4 | | 266 | | 84.4 | | 45 | | 88.2 | | 221 | | 83.7 |  |
| Don’t know | 11 | 3.4 | 1 | 1.8 | 10 | 3.7 | | 16 | | 5.1 | | 3 | | 5.9 | | 13 | | 4.9 |  |
| Missing | 10 | 3.1 | 1 | 1.8 | 9 | 3.4 | | 12 | | 3.8 | | 2 | | 3.9 | | 10 | | 3.8 |  |
| **Thinking about the last time you or your sexual partner were pregnant [of those who responded “Yes” to the previous question]:** | | | | | | |  | |  | |  | |  | |  | |  | |  |
| I intended to get pregnant/my partner pregnant | 13 | 40.6 | 0 | 0.0 | 13 | 41.9 | | 6 | | 28.6 | | 0 | | 0.0 | | 6 | | 30.0 |  |
| I didn’t think about the chance of getting pregnant/my partner pregnant | 6 | 18.8 | 1 | 100.0 | 5 | 16.1 | | 4 | | 19.1 | | 1 | | 100.0 | | 3 | | 15.0 |  |
| I did not mind if I/my partner got pregnant | 6 | 18.8 | 0 | 0.0 | 6 | 19.4 | | 3 | | 14.3 | | 0 | | 0.0 | | 3 | | 15.0 |  |
| I did not want to get myself/my partner pregnant | 7 | 21.9 | 0 | 0.0 | 7 | 22.6 | | 8 | | 38.1 | | 0 | | 0.0 | | 8 | | 40.0 |  |
| Missing | 0 | 0.0 | 0 | 0.0 | 0 | 0.0 | | 0 | | 0.0 | | 0 | | 0.0 | | 0 | | 0.0 |  |

**^a^** In this table, the analysis is based on the sample of those participants who reported to “have ever had vaginal and/or anal sex” (N=325 baseline sample, N=315 follow-up sample).

**eTable 7. Secondary outcome of** **sexual harassment taking place at further education settings**

|  | **Baseline** | | | | | | | | | | **12 Months Follow-up** | | | | | | |
| --- | --- | --- | --- | --- | --- | --- | --- | --- | --- | --- | --- | --- | --- | --- | --- | --- | --- |
|  | **Overall (N=1124)** | | | **Control (N=263)** | | | | **Intervention (N=861)** | | | **Overall (N=1139)** | | **Control (N=269)** | | **Intervention (N=870)** | | |
|  | **n** | **%** | | **n** | | **%** | | **n** | | **%** | **n** | **%** | **n** | **%** | **n** | **%** | |
| **How often have you experienced sexual harassment at 6th form/college?** | | | | | | | | | | | | | | | | |  |
| Never | 835 | 74.3 | | 216 | | 82.1 | | 619 | | 71.9 | 854 | 75.0 | 221 | 82.2 | 633 | 72.8 | |
| Once or twice | 129 | 11.5 | | 21 | | 8.0 | | 108 | | 12.5 | 121 | 10.6 | 15 | 5.6 | 106 | 12.2 | |
| About once a month | 18 | 1.6 | | 1 | | 0.4 | | 17 | | 2.0 | 8 | 0.7 | 1 | 0.4 | 7 | 0.8 | |
| 2 or 3 times a month | 6 | 0.5 | | 0 | | 0.0 | | 6 | | 0.7 | 6 | 0.5 | 2 | 0.7 | 4 | 0.5 | |
| About once a week | 7 | 0.6 | | 0 | | 0.0 | | 7 | | 0.8 | 5 | 0.4 | 0 | 0.0 | 5 | 0.6 | |
| More than once a week | 4 | 0.4 | | 1 | | 0.4 | | 3 | | 0.3 | 7 | 0.6 | 2 | 0.7 | 5 | 0.6 | |
| Missing | 125 | 11.1 | | 24 | | 9.1 | | 101 | | 11.7 | 138 | 12.1 | 28 | 10.4 | 110 | 12.6 | |
| **How often have you experienced sexual harassment in places other than 6th form/college?** | | | | | | | | | | | | | | | | |  |
| Never | 580 | 51.6 | | 149 | | 56.7 | | 431 | | 50.1 | 604 | 53.0 | 162 | 60.2 | 442 | 50.8 | |
| Once or twice | 226 | 20.1 | | 48 | | 18.3 | | 178 | | 20.7 | 243 | 21.3 | 47 | 17.5 | 196 | 22.5 | |
| About once a month | 78 | 6.9 | | 16 | | 6.1 | | 62 | | 7.2 | 62 | 5.4 | 10 | 3.7 | 52 | 6.0 | |
| 2 or 3 times a month | 63 | 5.6 | | 14 | | 5.3 | | 49 | | 5.7 | 52 | 4.6 | 9 | 3.3 | 43 | 4.9 | |
| About once a week | 36 | 3.2 | | 6 | | 2.3 | | 30 | | 3.5 | 19 | 1.7 | 9 | 3.3 | 10 | 1.1 | |
| More than once a week | 16 | 1.4 | | 7 | | 2.7 | | 9 | | 1.0 | 15 | 1.3 | 3 | 1.1 | 12 | 1.4 | |
| Missing | 125 | 11.1 | | 23 | | 8.7 | | 102 | | 11.8 | 144 | 12.6 | 29 | 10.8 | 115 | 13.2 | |
| **Have you ever been called offensive sexual names (e.g. slut, slag, bitch, fag, etc.) at 6th form/college?** | | | | | | | | | | | | | | | | |  |
| **By a male:** |  |  | |  | |  | |  | |  |  |  |  |  |  |  | |
| Never | 659 | 58.6 | | 187 | | 71.1 | | 472 | | 54.8 | 659 | 57.9 | 170 | 63.2 | 489 | 56.2 | |
| Once or twice | 204 | 18.1 | | 33 | | 12.6 | | 171 | | 19.9 | 185 | 16.2 | 35 | 13.0 | 150 | 17.2 | |
| About once a month | 37 | 3.3 | | 3 | | 1.1 | | 34 | | 3.9 | 36 | 3.2 | 6 | 2.2 | 30 | 3.4 | |
| 2 or 3 times a month | 28 | 2.5 | | 2 | | 0.8 | | 26 | | 3.0 | 32 | 2.8 | 5 | 1.9 | 27 | 3.1 | |
| About once a week | 27 | 2.4 | | 2 | | 0.8 | | 25 | | 2.9 | 30 | 2.6 | 9 | 3.3 | 21 | 2.4 | |
| More than once a week | 42 | 3.7 | | 13 | | 4.9 | | 29 | | 3.4 | 53 | 4.7 | 13 | 4.8 | 40 | 4.6 | |
| Missing | 127 | 11.3 | | 23 | | 8.8 | | 104 | | 12.1 | 144 | 12.6 | 31 | 11.5 | 113 | 13.0 | |
| **By a female:** | |  |  | |  | |  | |  | |  |  |  |  |  |  |  |
| Never | 746 | 66.4 | | 192 | | 73.0 | | 554 | | 64.3 | 748 | 65.7 | 174 | 64.7 | 574 | 66.0 | |
| Once or twice | 166 | 14.8 | | 31 | | 11.8 | | 135 | | 15.7 | 151 | 13.3 | 38 | 14.1 | 113 | 13.0 | |
| About once a month | 29 | 2.6 | | 2 | | 0.8 | | 27 | | 3.1 | 25 | 2.2 | 5 | 1.9 | 20 | 2.3 | |
| 2 or 3 times a month | 14 | 1.2 | | 3 | | 1.1 | | 11 | | 1.3 | 16 | 1.4 | 3 | 1.1 | 13 | 1.5 | |
| About once a week | 8 | 0.7 | | 1 | | 0.4 | | 7 | | 0.8 | 15 | 1.3 | 5 | 1.9 | 10 | 1.1 | |
| More than once a week | 28 | 2.5 | | 10 | | 3.8 | | 18 | | 2.1 | 28 | 2.5 | 11 | 4.1 | 17 | 2.0 | |
| Missing | 133 | 11.8 | | 24 | | 9.1 | | 109 | | 12.7 | 156 | 13.7 | 33 | 12.3 | 123 | 14.1 | |
| **By others that neither identify as male nor female:** | | | | | | | | | | |  |  |  |  |  |  |  |
| Never | 915 | 81.4 | | 222 | | 84.4 | | 693 | | 80.5 | 909 | 79.8 | 225 | 83.6 | 684 | 78.6 | |
| Once or twice | 49 | 4.4 | | 10 | | 3.8 | | 39 | | 4.5 | 53 | 4.7 | 8 | 3.0 | 45 | 5.2 | |
| About once a month | 8 | 0.7 | | 1 | | 0.4 | | 7 | | 0.8 | 4 | 0.4 | 1 | 0.4 | 3 | 0.3 | |
| 2 or 3 times a month | 5 | 0.4 | | 3 | | 1.1 | | 2 | | 0.2 | 4 | 0.4 | 1 | 0.4 | 3 | 0.3 | |
| About once a week | 4 | 0.4 | | 1 | | 0.4 | | 3 | | 0.3 | 4 | 0.4 | 0 | 0.0 | 4 | 0.5 | |
| More than once a week | 6 | 0.5 | | 2 | | 0.8 | | 4 | | 0.5 | 5 | 0.4 | 1 | 0.4 | 4 | 0.5 | |
| Missing | 137 | 12.2 | | 24 | | 9.1 | | 113 | | 13.1 | 160 | 14.0 | 33 | 12.3 | 127 | 14.6 | |
| **Have you ever unwantedly been sexually touched, groped or kissed by someone whilst in 6th form/college?** | | | | | | | | | | | | | | | | |  |
| No, never | 913 | 81.2 | | 223 | | 84.8 | | 690 | | 80.1 | 922 | 80.9 | 231 | 85.9 | 691 | 79.4 | |
| Once or twice | 69 | 6.1 | | 14 | | 5.3 | | 55 | | 6.4 | 64 | 5.6 | 7 | 2.6 | 57 | 6.6 | |
| About once a month | 8 | 0.7 | | 1 | | 0.4 | | 7 | | 0.8 | 2 | 0.2 | 0 | 0.0 | 2 | 0.2 | |
| 2 or 3 times a month | 3 | 0.3 | | 0 | | 0.0 | | 3 | | 0.3 | 3 | 0.3 | 0 | 0.0 | 3 | 0.3 | |
| About once a week | 3 | 0.3 | | 0 | | 0.0 | | 3 | | 0.3 | 0 | 0.0 | 0 | 0.0 | 0 | 0.0 | |
| More than once a week | 2 | 0.2 | | 2 | | 0.8 | | 0 | | 0.0 | 7 | 0.6 | 3 | 1.1 | 4 | 0.5 | |
| Missing | 126 | 11.2 | | 23 | | 8.7 | | 103 | | 12.0 | 141 | 12.4 | 28 | 10.4 | 113 | 13.0 | |
| **Have you ever seen pornographic content that you didn’t want to whilst in 6th form/college?** | | | | | | | | | | | | | | | | |  |
| No, never | 843 | 75.0 | | 214 | | 81.4 | | 629 | | 73.1 | 850 | 74.6 | 214 | 79.6 | 636 | 73.1 | |
| Once or twice | 121 | 10.8 | | 20 | | 7.6 | | 101 | | 11.7 | 118 | 10.4 | 19 | 7.1 | 99 | 11.4 | |
| About once a month | 19 | 1.7 | | 4 | | 1.5 | | 15 | | 1.7 | 11 | 1.0 | 3 | 1.1 | 8 | 0.9 | |
| 2 or 3 times a month | 6 | 0.5 | | 1 | | 0.4 | | 5 | | 0.6 | 10 | 0.9 | 3 | 1.1 | 7 | 0.8 | |
| About once a week | 6 | 0.5 | | 2 | | 0.8 | | 4 | | 0.5 | 2 | 0.2 | 0 | 0.0 | 2 | 0.2 | |
| More than once a week | 23 | 2.0 | | 22 | | 8.4 | | 1 | | 0.1 | 6 | 0.5 | 1 | 0.4 | 5 | 0.6 | |
| Missing | 108 | 9.6 | | 2 | | 0.8 | | 106 | | 12.3 | 142 | 12.5 | 29 | 10.8 | 113 | 13.0 | |
| **Has anyone at your 6th form/college ever sent, forwarded or shared a sexually explicit image or video of you to other people, without asking you?** | | | | | | | | | | | | | | | | |  |
| No, never | 948 | 84.3 | | 230 | | 87.5 | | 718 | | 83.4 | 951 | 83.5 | 232 | 86.2 | 719 | 82.6 | |
| Yes, once | 34 | 3.0 | | 6 | | 2.3 | | 28 | | 3.3 | 32 | 2.8 | 6 | 2.2 | 26 | 3.0 | |
| Yes, more than once | 17 | 1.5 | | 5 | | 1.9 | | 12 | | 1.4 | 23 | 2.0 | 5 | 1.9 | 18 | 2.1 | |
| Missing | 125 | 11.1 | | 22 | | 8.4 | | 103 | | 12.0 | 133 | 11.7 | 26 | 9.7 | 107 | 12.3 | |

**eTable 8. Secondary outcome of** **relationship with partner** **(sCADRI)**

|  | **Baseline** | | | | | | **12 Months Follow-up** | | | | | |
| --- | --- | --- | --- | --- | --- | --- | --- | --- | --- | --- | --- | --- |
|  | **Overall (N=643)** | | **Control (N=125)** | | **Intervention (N=518)** | | **Overall (N=674)** | | **Control (N=125)** | | **Intervention (N=549)** | |
|  | **n** | **%** | **n** | **%** | **n** | **%** | **n** | **%** | **n** | **%** | **n** | **%** |
| **My partner**  **Spoke to me in a hostile/mean tone of voice:** | | | | | | |  |  |  |  |  |  |
| Never | 378 | 58.8 | 74 | 59.2 | 304 | 58.7 | 411 | 61.0 | 78 | 62.4 | 333 | 60.7 |
| Rarely | 130 | 20.2 | 26 | 20.8 | 104 | 20.1 | 125 | 18.5 | 25 | 20.0 | 100 | 18.2 |
| Sometimes | 73 | 11.4 | 13 | 10.4 | 60 | 11.6 | 80 | 11.9 | 13 | 10.4 | 67 | 12.2 |
| Often | 33 | 5.1 | 6 | 4.8 | 27 | 5.2 | 35 | 5.2 | 7 | 5.6 | 28 | 5.1 |
| N/A | 26 | 4.0 | 5 | 4.0 | 21 | 4.1 | 21 | 3.1 | 1 | 0.8 | 20 | 3.6 |
| Missing | 3 | 0.5 | 1 | 0.8 | 2 | 0.4 | 2 | 0.3 | 1 | 0.8 | 1 | 0.2 |
| **Insulted me with put-downs:** | | | | | |  |  |  |  |  |  |  |
| Never | 452 | 70.3 | 86 | 68.8 | 366 | 70.7 | 483 | 71.7 | 92 | 73.6 | 391 | 71.2 |
| Rarely | 83 | 12.9 | 15 | 12.0 | 68 | 13.1 | 76 | 11.3 | 18 | 14.4 | 58 | 10.6 |
| Sometimes | 64 | 10.0 | 15 | 12.0 | 49 | 9.5 | 64 | 9.5 | 10 | 8.0 | 54 | 9.8 |
| Often | 14 | 2.2 | 3 | 2.4 | 11 | 2.1 | 27 | 4.0 | 3 | 2.4 | 24 | 4.4 |
| N/A | 27 | 4.2 | 5 | 4.0 | 22 | 4.2 | 22 | 3.3 | 1 | 0.8 | 21 | 3.8 |
| Missing | 3 | 0.5 | 1 | 0.8 | 2 | 0.4 | 2 | 0.3 | 1 | 0.8 | 1 | 0.2 |
| **Said things to my friends about me to turn them against me:** | | | | | | |  |  |  |  |  |  |
| Never | 535 | 83.2 | 110 | 88.0 | 425 | 82.0 | 546 | 81.0 | 99 | 79.2 | 447 | 81.4 |
| Rarely | 31 | 4.8 | 4 | 3.2 | 27 | 5.2 | 44 | 6.5 | 10 | 8.0 | 34 | 6.2 |
| Sometimes | 29 | 4.5 | 4 | 3.2 | 25 | 4.8 | 34 | 5.0 | 8 | 6.4 | 26 | 4.7 |
| Often | 18 | 2.8 | 1 | 0.8 | 17 | 3.3 | 24 | 3.6 | 6 | 4.8 | 18 | 3.3 |
| N/A | 27 | 4.2 | 5 | 4.0 | 22 | 4.2 | 24 | 3.6 | 1 | 0.8 | 23 | 4.2 |
| Missing | 3 | 0.5 | 1 | 0.8 | 2 | 0.4 | 2 | 0.3 | 1 | 0.8 | 1 | 0.2 |
| **Kicked, hit, or punched me:** | | | | | |  |  |  |  |  |  |  |
| Never | 567 | 88.2 | 109 | 87.2 | 458 | 88.4 | 598 | 88.7 | 109 | 87.2 | 489 | 89.1 |
| Rarely | 29 | 4.5 | 6 | 4.8 | 23 | 4.4 | 32 | 4.7 | 10 | 8.0 | 22 | 4.0 |
| Sometimes | 8 | 1.2 | 1 | 0.8 | 7 | 1.4 | 11 | 1.6 | 0 | 0.0 | 11 | 2.0 |
| Often | 7 | 1.1 | 2 | 1.6 | 5 | 1.0 | 10 | 1.5 | 2 | 1.6 | 8 | 1.5 |
| N/A | 27 | 4.2 | 5 | 4.0 | 22 | 4.2 | 19 | 2.8 | 2 | 1.6 | 17 | 3.1 |
| Missing | 5 | 0.8 | 2 | 1.6 | 3 | 0.6 | 4 | 0.6 | 2 | 1.6 | 2 | 0.4 |
| **Slapped or pulled my hair:** | | | | | |  |  |  |  |  |  |  |
| Never | 566 | 88.0 | 110 | 88.0 | 456 | 88.0 | 604 | 89.6 | 112 | 89.6 | 492 | 89.6 |
| Rarely | 21 | 3.3 | 5 | 4.0 | 16 | 3.1 | 28 | 4.2 | 8 | 6.4 | 20 | 3.6 |
| Sometimes | 11 | 1.7 | 1 | 0.8 | 10 | 1.9 | 13 | 1.9 | 1 | 0.8 | 12 | 2.2 |
| Often | 11 | 1.7 | 2 | 1.6 | 9 | 1.7 | 10 | 1.5 | 2 | 1.6 | 8 | 1.5 |
| N/A | 30 | 4.7 | 6 | 4.8 | 24 | 4.6 | 17 | 2.5 | 1 | 0.8 | 16 | 2.9 |
| Missing | 4 | 0.6 | 1 | 0.8 | 3 | 0.6 | 2 | 0.3 | 1 | 0.8 | 1 | 0.2 |
| **Threatened to hurt me:** | | | | | |  |  |  |  |  |  |  |
| Never | 570 | 88.6 | 111 | 88.8 | 459 | 88.6 | 614 | 91.1 | 117 | 93.6 | 497 | 90.5 |
| Rarely | 22 | 3.4 | 4 | 3.2 | 18 | 3.5 | 15 | 2.2 | 0 | 0.0 | 15 | 2.7 |
| Sometimes | 9 | 1.4 | 1 | 0.8 | 8 | 1.5 | 17 | 2.5 | 4 | 3.2 | 13 | 2.4 |
| Often | 9 | 1.4 | 2 | 1.6 | 7 | 1.4 | 8 | 1.2 | 2 | 1.6 | 6 | 1.1 |
| N/A | 27 | 4.2 | 5 | 4.0 | 22 | 4.2 | 18 | 2.7 | 1 | 0.8 | 17 | 3.1 |
| Missing | 6 | 0.9 | 2 | 1.6 | 4 | 0.8 | 2 | 0.3 | 1 | 0.8 | 1 | 0.2 |
| **Threatened to hit or throw something at me:** | | | | | |  |  |  |  |  |  |  |
| Never | 565 | 87.9 | 110 | 88.0 | 455 | 87.8 | 606 | 89.9 | 113 | 90.4 | 493 | 89.8 |
| Rarely | 27 | 4.2 | 7 | 5.6 | 20 | 3.9 | 26 | 3.9 | 3 | 2.4 | 23 | 4.2 |
| Sometimes | 11 | 1.7 | 0 | 0.0 | 11 | 2.1 | 14 | 2.1 | 4 | 3.2 | 10 | 1.8 |
| Often | 10 | 1.6 | 2 | 1.6 | 8 | 1.5 | 8 | 1.2 | 2 | 1.6 | 6 | 1.1 |
| N/A | 26 | 4.0 | 5 | 4.0 | 21 | 4.1 | 18 | 2.7 | 2 | 1.6 | 16 | 2.9 |
| Missing | 4 | 0.6 | 1 | 0.8 | 3 | 0.6 | 2 | 0.3 | 1 | 0.8 | 1 | 0.2 |
| **Spread rumours about me:** | | | | | |  |  |  |  |  |  |  |
| Never | 529 | 82.3 | 106 | 84.8 | 423 | 81.7 | 555 | 82.3 | 104 | 83.2 | 451 | 82.1 |
| Rarely | 38 | 5.9 | 3 | 2.4 | 35 | 6.8 | 37 | 5.5 | 10 | 8.0 | 27 | 4.9 |
| Sometimes | 21 | 3.3 | 7 | 5.6 | 14 | 2.7 | 34 | 5.0 | 1 | 0.8 | 33 | 6.0 |
| Often | 23 | 3.6 | 2 | 1.6 | 21 | 4.1 | 26 | 3.9 | 8 | 6.4 | 18 | 3.3 |
| N/A | 27 | 4.2 | 6 | 4.8 | 21 | 4.1 | 19 | 2.8 | 1 | 0.8 | 18 | 3.3 |
| Missing | 5 | 0.8 | 1 | 0.8 | 4 | 0.8 | 3 | 0.4 | 1 | 0.8 | 2 | 0.4 |
| **Touched me sexually when I didn't want them to:** | | | | | | |  |  |  |  |  |  |
| Never | 539 | 83.8 | 106 | 84.8 | 433 | 83.6 | 590 | 87.5 | 116 | 92.8 | 474 | 86.3 |
| Rarely | 37 | 5.8 | 8 | 6.4 | 29 | 5.6 | 24 | 3.6 | 2 | 1.6 | 22 | 4.0 |
| Sometimes | 22 | 3.4 | 2 | 1.6 | 20 | 3.9 | 28 | 4.2 | 2 | 1.6 | 26 | 4.7 |
| Often | 14 | 2.2 | 3 | 2.4 | 11 | 2.1 | 12 | 1.8 | 3 | 2.4 | 9 | 1.6 |
| N/A | 26 | 4.0 | 5 | 4.0 | 21 | 4.1 | 18 | 2.7 | 1 | 0.8 | 17 | 3.1 |
| Missing | 5 | 0.8 | 1 | 0.8 | 4 | 0.8 | 2 | 0.3 | 1 | 0.8 | 1 | 0.2 |
| **Forced me to have sex when I didn't want to:** | | | | | |  |  |  |  |  |  |  |
| Never | 573 | 89.1 | 111 | 88.8 | 462 | 89.2 | 624 | 92.6 | 119 | 95.2 | 505 | 92.0 |
| Rarely | 13 | 2.0 | 2 | 1.6 | 11 | 2.1 | 13 | 1.9 | 1 | 0.8 | 12 | 2.2 |
| Sometimes | 14 | 2.2 | 0 | 0.0 | 14 | 2.7 | 6 | 0.9 | 0 | 0.0 | 6 | 1.1 |
| Often | 9 | 1.4 | 3 | 2.4 | 6 | 1.2 | 6 | 0.9 | 2 | 1.6 | 4 | 0.7 |
| N/A | 28 | 4.4 | 7 | 5.6 | 21 | 4.1 | 23 | 3.4 | 2 | 1.6 | 21 | 3.8 |
| Missing | 6 | 0.9 | 2 | 1.6 | 4 | 0.8 | 2 | 0.3 | 1 | 0.8 | 1 | 0.2 |
| **I spoke to my partner in a hostile/mean tone:** | | | | | |  |  |  |  |  |  |  |
| Never | 442 | 68.7 | 88 | 70.4 | 354 | 68.3 | 480 | 71.2 | 87 | 69.6 | 393 | 71.6 |
| Rarely | 125 | 19.4 | 22 | 17.6 | 103 | 19.9 | 121 | 18.0 | 26 | 20.8 | 95 | 17.3 |
| Sometimes | 40 | 6.2 | 8 | 6.4 | 32 | 6.2 | 49 | 7.3 | 9 | 7.2 | 40 | 7.3 |
| Often | 8 | 1.2 | 1 | 0.8 | 7 | 1.4 | 4 | 0.6 | 1 | 0.8 | 3 | 0.5 |
| N/A | 24 | 3.7 | 5 | 4.0 | 19 | 3.7 | 16 | 2.4 | 1 | 0.8 | 15 | 2.7 |
| Missing | 4 | 0.6 | 1 | 0.8 | 3 | 0.6 | 4 | 0.6 | 1 | 0.8 | 3 | 0.5 |
| **I insulted my partner with put-downs:** | | | | | |  |  |  |  |  |  |  |
| Never | 548 | 85.2 | 106 | 84.8 | 442 | 85.3 | 576 | 85.5 | 106 | 84.8 | 470 | 85.6 |
| Rarely | 50 | 7.8 | 11 | 8.8 | 39 | 7.5 | 64 | 9.5 | 14 | 11.2 | 50 | 9.1 |
| Sometimes | 13 | 2.0 | 0 | 0.0 | 13 | 2.5 | 10 | 1.5 | 3 | 2.4 | 7 | 1.3 |
| Often | 2 | 0.3 | 1 | 0.8 | 1 | 0.2 | 4 | 0.6 | 0 | 0.0 | 4 | 0.7 |
| N/A | 23 | 3.6 | 5 | 4.0 | 18 | 3.5 | 15 | 2.2 | 1 | 0.8 | 14 | 2.6 |
| Missing | 7 | 1.1 | 2 | 1.6 | 5 | 1.0 | 5 | 0.7 | 1 | 0.8 | 4 | 0.7 |
| **I said things to my partner's friends to turn them against him/her:** | | | | | | |  |  |  |  |  |  |
| Never | 596 | 92.7 | 118 | 94.4 | 478 | 92.3 | 627 | 93.0 | 118 | 94.4 | 509 | 92.7 |
| Rarely | 14 | 2.2 | 1 | 0.8 | 13 | 2.5 | 16 | 2.4 | 4 | 3.2 | 12 | 2.2 |
| Sometimes | 4 | 0.6 | 0 | 0.0 | 4 | 0.8 | 7 | 1.0 | 1 | 0.8 | 6 | 1.1 |
| Often | 0 | 0.0 | 0 | 0.0 | 0 | 0.0 | 3 | 0.4 | 0 | 0.0 | 3 | 0.5 |
| N/A | 24 | 3.7 | 5 | 4.0 | 19 | 3.7 | 15 | 2.2 | 1 | 0.8 | 14 | 2.6 |
| Missing | 5 | 0.8 | 1 | 0.8 | 4 | 0.8 | 6 | 0.9 | 1 | 0.8 | 5 | 0.9 |
| **I kicked, hit, or punched my partner:** | | | | | |  |  |  |  |  |  |  |
| Never | 600 | 93.3 | 115 | 92.0 | 485 | 93.6 | 639 | 94.8 | 120 | 96.0 | 519 | 94.5 |
| Rarely | 7 | 1.1 | 3 | 2.4 | 4 | 0.8 | 10 | 1.5 | 3 | 2.4 | 7 | 1.3 |
| Sometimes | 1 | 0.2 | 0 | 0.0 | 1 | 0.2 | 1 | 0.1 | 0 | 0.0 | 1 | 0.2 |
| Often | 3 | 0.5 | 1 | 0.8 | 2 | 0.4 | 3 | 0.4 | 0 | 0.0 | 3 | 0.5 |
| N/A | 25 | 3.9 | 5 | 4.0 | 20 | 3.9 | 15 | 2.2 | 1 | 0.8 | 14 | 2.6 |
| Missing | 7 | 1.1 | 1 | 0.8 | 6 | 1.2 | 6 | 0.9 | 1 | 0.8 | 5 | 0.9 |
| **I slapped or pulled my partner's hair:** | | | | | |  |  |  |  |  |  |  |
| Never | 597 | 92.8 | 116 | 92.8 | 481 | 92.9 | 642 | 95.3 | 122 | 97.6 | 520 | 94.7 |
| Rarely | 6 | 0.9 | 2 | 1.6 | 4 | 0.8 | 9 | 1.3 | 1 | 0.8 | 8 | 1.5 |
| Sometimes | 6 | 0.9 | 1 | 0.8 | 5 | 1.0 | 1 | 0.1 | 0 | 0.0 | 1 | 0.2 |
| Often | 2 | 0.3 | 0 | 0.0 | 2 | 0.4 | 3 | 0.4 | 0 | 0.0 | 3 | 0.5 |
| N/A | 23 | 3.6 | 5 | 4.0 | 18 | 3.5 | 15 | 2.2 | 1 | 0.8 | 14 | 2.6 |
| Missing | 9 | 1.4 | 1 | 0.8 | 8 | 1.5 | 4 | 0.6 | 1 | 0.8 | 3 | 0.5 |
| **I threatened to hurt my partner:** | | | | | |  |  |  |  |  |  |  |
| Never | 609 | 94.7 | 120 | 96.0 | 489 | 94.4 | 645 | 95.7 | 122 | 97.6 | 523 | 95.3 |
| Rarely | 2 | 0.3 | 0 | 0.0 | 2 | 0.4 | 4 | 0.6 | 0 | 0.0 | 4 | 0.7 |
| Sometimes | 2 | 0.3 | 0 | 0.0 | 2 | 0.4 | 1 | 0.1 | 0 | 0.0 | 1 | 0.2 |
| Often | 0 | 0.0 | 0 | 0.0 | 0 | 0.0 | 2 | 0.3 | 0 | 0.0 | 2 | 0.4 |
| N/A | 22 | 3.4 | 4 | 3.2 | 18 | 3.5 | 15 | 2.2 | 1 | 0.8 | 14 | 2.6 |
| Missing | 8 | 1.2 | 1 | 0.8 | 7 | 1.4 | 7 | 1.0 | 2 | 1.6 | 5 | 0.9 |
| **I threatened to hit or throw something at my partner:** | | | | | | |  |  |  |  |  |  |
| Never | 603 | 93.8 | 117 | 93.6 | 486 | 93.8 | 641 | 95.1 | 120 | 96.0 | 521 | 94.9 |
| Rarely | 8 | 1.2 | 2 | 1.6 | 6 | 1.2 | 10 | 1.5 | 3 | 2.4 | 7 | 1.3 |
| Sometimes | 1 | 0.2 | 0 | 0.0 | 1 | 0.2 | 0 | 0.0 | 0 | 0.0 | 0 | 0.0 |
| Often | 0 | 0.0 | 0 | 0.0 | 0 | 0.0 | 3 | 0.4 | 0 | 0.0 | 3 | 0.5 |
| N/A | 23 | 3.6 | 5 | 4.0 | 18 | 3.5 | 15 | 2.2 | 1 | 0.8 | 14 | 2.6 |
| Missing | 8 | 1.2 | 1 | 0.8 | 7 | 1.4 | 5 | 0.7 | 1 | 0.8 | 4 | 0.7 |
| **I spread rumours about my partner:** | | | | | |  |  |  |  |  |  |  |
| Never | 596 | 92.7 | 113 | 90.4 | 483 | 93.2 | 638 | 94.7 | 119 | 95.2 | 519 | 94.5 |
| Rarely | 9 | 1.4 | 3 | 2.4 | 6 | 1.2 | 13 | 1.9 | 4 | 3.2 | 9 | 1.6 |
| Sometimes | 5 | 0.8 | 2 | 1.6 | 3 | 0.6 | 1 | 0.1 | 0 | 0.0 | 1 | 0.2 |
| Often | 0 | 0.0 | 0 | 0.0 | 0 | 0.0 | 3 | 0.4 | 0 | 0.0 | 3 | 0.5 |
| N/A | 24 | 3.7 | 5 | 4.0 | 19 | 3.7 | 15 | 2.2 | 1 | 0.8 | 14 | 2.6 |
| Missing | 9 | 1.4 | 2 | 1.6 | 7 | 1.4 | 4 | 0.6 | 1 | 0.8 | 3 | 0.5 |
| **I touched my partner sexually when they didn't want me to:** | | | | | | |  |  |  |  |  |  |
| Never | 601 | 93.5 | 116 | 92.8 | 485 | 93.6 | 647 | 96.0 | 122 | 97.6 | 525 | 95.6 |
| Rarely | 6 | 0.9 | 2 | 1.6 | 4 | 0.8 | 3 | 0.4 | 1 | 0.8 | 2 | 0.4 |
| Sometimes | 0 | 0.0 | 0 | 0.0 | 0 | 0.0 | 0 | 0.0 | 0 | 0.0 | 0 | 0.0 |
| Often | 0 | 0.0 | 0 | 0.0 | 0 | 0.0 | 2 | 0.3 | 0 | 0.0 | 2 | 0.4 |
| N/A | 24 | 3.7 | 5 | 4.0 | 19 | 3.7 | 15 | 2.2 | 1 | 0.8 | 14 | 2.6 |
| Missing | 12 | 1.9 | 2 | 1.6 | 10 | 1.9 | 7 | 1.0 | 1 | 0.8 | 6 | 1.1 |
| **I forced my partner to have sex when they didn't want to:** | | | | | | |  |  |  |  |  |  |
| Never | 601 | 93.5 | 114 | 91.2 | 487 | 94.0 | 650 | 96.4 | 122 | 97.6 | 528 | 96.2 |
| Rarely | 0 | 0.0 | 0 | 0.0 | 0 | 0.0 | 0 | 0.0 | 0 | 0.0 | 0 | 0.0 |
| Sometimes | 0 | 0.0 | 0 | 0.0 | 0 | 0.0 | 0 | 0.0 | 0 | 0.0 | 0 | 0.0 |
| Often | 1 | 0.2 | 1 | 0.8 | 0 | 0.0 | 2 | 0.3 | 0 | 0.0 | 2 | 0.4 |
| N/A | 26 | 4.0 | 7 | 5.6 | 19 | 3.7 | 16 | 2.4 | 2 | 1.6 | 14 | 2.6 |
| Missing | 15 | 2.3 | 3 | 2.4 | 12 | 2.3 | 6 | 0.9 | 1 | 0.8 | 5 | 0.9 |

Percentage calculation for each item of the scale includes missing observations in the denominator. In the table heading, N=643 (314+329) for the sample at baseline is the sample size based on a “Yes” response to the question, “Have you ever dated or been in a relationship” in Table 4 above. However, this sample size is not applicable for each item of the sCADRI because of the missing observations. For each item, the applicable sample size may vary (from 639 to 628). N=674 (309+365) for the sample at follow-up is the sample size based on a “Yes” response to the question, “Have you ever dated or been in a relationship at follow-up”. However, this sample size is also not applicable for each item of the sCADRI because of the missing observations. For each item, the applicable sample size may vary.

**eTable 9. Secondary outcome assessed in intervention group only: awareness and potential use of the service**

| **N=870** | **n** | **%** |
| --- | --- | --- |
| **Are you aware that your school / college has had a sexual health service 2 days?** | | |
| Yes | 208 | 23.9 |
| No | 385 | 44.3 |
| Missing | 277 | 31.8 |
| **How were you made aware of the service (select all that apply)? [of n=208 who responded “Yes” to the previous question]** | | |
| Tutor | 138 | 66.4 |
| Posters | 59 | 28.4 |
| Screens | 14 | 6.7 |
| Email | 95 | 45.7 |
| Wellbeing team | 36 | 17.3 |
| Social media | 4 | 1.9 |
| Word of mouth | 42 | 20.2 |
| Other | 1 | 0.5 |
| **If you had been aware of the service, is it something you would have used if needed? [of n=385 who responded “No” to the previous question]** | | |
| Yes | 117 | 30.4 |
| No | 266 | 69.1 |
| Missing | 2 | 0.5 |
| **During the last academic year, did you have any instances where you may have needed to use these services??** | | |
| Yes | 62 | 7.1 |
| No | 530 | 60.9 |
| Missing | 278 | 32.0 |
| **Did you attend the service on site? [of n=62 who responded “Yes” to the previous question]** | | |
| Yes | 11 | 17.7 |
| No | 50 | 80.6 |
| Missing | 1 | 1.6 |
| **Did you get what you wanted from the drop-in service? [of n=11 who responded “Yes” to the previous question]** | | |
| Yes | 11 | 100.0 |
| **What provision were you given (select all that apply)? [of n=11 who responded “Yes” to the previous question]** | | |
| Advice | 3 | 27.3 |
| Condoms | 8 | 72.7 |
| STI tests | 1 | 9.1 |
| Pregnancy test | 2 | 18.2 |
| **Have any school / college staff members helped you with advice or support with an issue relating to sexual health or dating and relationship violence during the 2021-22 academic year?** | | |
| Yes | 26 | 3.0 |
| No - not needed | 517 | 59.4 |
| No - I did not feel comfortable asking staff for support | 45 | 5.2 |
| Missing | 282 | 32.4 |
| **Do you feel that the staff member had enough knowledge to be able to support you? [of n=26 who responded “Yes” to the previous question]** | | |
| Yes | 23 | 88.5 |
| No | 3 | 11.5 |
