# Supplementary material for: Sexual health and healthy relationships for Further Education (SaFE) in Wales and England: results from a pilot cluster randomised controlled trial
Source: BMJ Open. 2024 Dec 20;14(12):e091355. doi: 10.1136/bmjopen-2024-091355 (PMC11667339; doi:10.1136/bmjopen-2024-091355)
Supplement: Supplementary file 2 [file bmjopen-2024-091355.reviewer_comments.pdf]

PEER REVIEW HISTORY

BMJ Open publishes all reviews undertaken for accepted manuscripts. Reviewers are asked to complete a checklist review form and are provided with free text boxes to elaborate on their assessment. These free text comments are reproduced below.

ARTICLE DETAILS

Title (Provisional)

Sexual health and healthy relationships for Further Education (SaFE) in Wales and England: results from a pilot cluster randomised controlled trial

Authors

Williams-Thomas, Rhys; Townson, Julia; Lewis, Ruth; Copeland, Lauren; Madan, Jason; Melendez-Torres, G.J; Lugg-Widger, Fiona V; Pallmann, Philip; Riaz, Muhammad; Brown, Rachel; Bonell, Chris; Morgan, Gemma S.; White, James; Young, Honor

---

VERSION 1 - REVIEW

**Reviewer**	**1**
**Name**	**Habtu, Yitagesu**
**Affiliation**	**Addis Ababa University, School of Public health**
**Date**	**06-Aug-2024**
**COI**	**I have no conflict of interest to declare.**

I would like to thank the editor for allowing me to review the manuscript. The paper examined the acceptability of implementing, trialling and estimating the cost of Sexual health and healthy relationships for Further Education (SaFE) intervention. I believe the findings provide additional insights for policymakers and the scientific community. The paper is well writen, and readable. However, I believe the authors are required to address a few comments and /or otherwise clarify more to improve the manuscript.

Methods

Randomization ?

• In your study population, "FE setting retention was incentivized with a £500 payment on completion of the study." Was this incentive communicated to participants before the intervention started, or only after their participation had ended?

• How did you control for baseline differences that could arise due to random chance, and the smaller size of the control group, 3 :1? What measures did you take to address potential confounders resulting from this unequal allocation?

• You mentioned, “The allocation was stratified by country and type of setting.” Was there a strategy in place to prevent contact, including through social media, between students or teachers in the control groups and those in the intervention groups?

• You stated, “It was not possible for study participants (students), teachers, trial managers, the intervention delivery team, or researchers involved in the process evaluation to be blind to intervention status.” Considering that teachers and students were aware of their allocation, how did you address the possibility that this awareness could alter their behavior and potentially influence the outcomes?

• How did you ensure consistency in the delivery of the intervention across different settings, accounting for variations in training, adherence to the intervention protocol, and contextual factors (e.g., differences in countries and types of settings)?

• What measures did you take to assess external factors, such as concurrent interventions or changes within the country or setting, that might have occurred during your 12-month intervention period?

• While stratification by country and type of setting helps balance certain factors, what strategies did you employ to assess any residual imbalance within strata or address unmeasured factors?

Results

• Given that your study employed a mixed-methods approach, I think it should be systematically integrated in the way that your qualitative adds something on your quantitative findings ?

**Reviewer**	**2**
**Name**	**Dlamini, Siyabonga**
**Affiliation**	**University of KwaZulu-Natal, Public Health Medicine**
**Date**	**17-Aug-2024**
**COI**	**None**

Thank you for taking the time to report on this area of research targeting young people’s sexual health. Young people are often a group that is difficult to reach and deliver sexual health services to. This study has a potential to answer some of the pertinent questions on how to effectively package these services targeted to this particular group. However, there are important considerations that the authors have to account for before the article can be published, in my view.

Review of the ar-cle: Sexual health and healthy rela-onships for Further Educa-on (SaFE): results from a pilot cluster randomised controlled trial

Thank you for the opportunity to contribute posi3vely to the review and be8erment of this ar3cle. The authors are tackling an important area of research targe3ng young people’s sexual health. Young people are o?en a group that is difficult to reach and deliver sexual health services to. This study has a poten3al to answer some of the per3nent ques3ons on how to effec3vely package these services targeted to this par3cular group. However, there are important considera3ons that the authors have to account for before the ar3cle can be published, in my view.

I have structured my report into two parts. The first part addresses broad concerns about the ar3cle in terms of the structure, methods, and the internal cohesion of the ar3cle. The second part highlights the granular issues to be a8ended to by sec3on.

1. **Part 1: Broad concerns about the ar-cle**

The 3tle of the ar3cle suggests the authors would report about the cluster randomised controlled trial which is a quan3ta3ve study. However, the authors also report on the qualita3ve data they collected during the course of the study. Although it is not a bad idea to report the results of these two approaches, it would be best to report them separately. The challenge with the current ar3cle is that both the methods and results of the two study approaches (qualita3ve and quan3ta3ve) are not sufficiently reported. Important details are missing in these two important sec3ons. It would be best for the authors to write two papers, where one paper presents the findings of the qualita3ve study and another the quan3ta3ve results.

2. **Part 2: Feedback on the specific sec-ons of the ar-cle**

Abstract

Results

It is unclear how long the interven3on took before the follow-up measurements were done. The authors only men3on the 12-month follow-up. In the text, the authors only clarify that it is 12 months a?er the baseline. That is not enough, because they do not report on the length of the interven3on nor the length of 3me to follow-up/post-interven3on measurements a?er the implementa3on of the interven3on.

Introduc-on

Page 5, line 7: "In 2022, NICE recommended…" – **The authors should write this acronym in full first before using the acronym.**

Line 20: "…se[ngs have a transient student popula3on,…" – **In what way?**

Line 22: "…as range of programmes and services offered." – **The authors should provide a source(s).**

Methods

Page 6, line 6: "Study design and par3cipants" – **More descrip-on of the study design is needed.**

Line 10: "…extension for pilot and feasibility trials." – **This point needs to be made explicitly. It is unclear that the study was a pilot un-l later.**

Lines 12-14: "…and all interested were visited to discuss the study in more detail and agree a research contract." – **To what extent did this approach introduce bias to this study?**

Data Collec-on

Page 7, line 47: "…were taken 12-months post baseline." – **What was the -me interval between the comple-on of the interven-on and the post-interven-on survey? How long aQer the interven-on was the second set of measurements taken?**

Lines 56-60: "Structured observa3ons of staff training (n=1 per se[ng), focus groups with students (n=2 per se[ng) and telephone interviews with trained FE staff (n=4 per se[ng) and onsite sexual health service staff (n=1 per se[ng) examined interven3on…" – **I encourage the authors to report the two study approaches separately to sufficiently address the methodological considera-ons of the two study approaches.**

Ethics and Consent

Page 8, lines 14-16: "Students were provided with informa3on sheets and consent forms, and, following informed consent,…" – **It is unclear to me how consent was sought among students younger than 18 years old. Was the parents' approval sought for these students?**

Line 18: "…ques3onnaire were offered entry into a prize draw to win an iPad." – **I am wondering if this approach was not seen as an undue inducement.**

Lines 18-20: "The baseline survey occurred in Sep/Oct 2021 and follow-up 12 months post baseline (Sep/Oct 2022)." – **This statement is not part of ethics discussion.**

Line 22: "Trained fieldworkers a8ended social areas and lessons to invite students to par3cipate." – **Is this not part of the recruitment strategy?**

Line 24: "Ethical approval was…" – **Is it not a requirement in this context to obtain gatekeeper permission from the par-cipa-ng ins-tu-ons?**

Line 60: "…4Ps…" – **The authors should discuss this (4Ps) further. What do these 4Ps represent?**

Page 9, Line 5: "…at low price…" – **Grammar? Should this not read, "... at a low price..."?**

PPI

Page 11, Lines 11-15: "This study builds on 15 months of work with over 2000 students and 200 staff from six FE se[ngs, 12 sexual health staff and an advisory group of 16-21 year olds (ALPHA) to explore which components should be combined into an interven3on." – **Does this mean that all these individuals were part of this study?**

Outcome measures

Lines 33-35: "…of the pilot cRCT was whether progression to a phase III RCT is jus3fied in terms of progression criteria…" – **The authors need to explicitly men-on the design of this study earlier. It is not discussed explicitly in the methods sec-on.**

Line 44: "We also examined the indica3ve primary outcomes of a future phase III trial." – **It seems like this part is outside the scope of the study. Relevance?**

Analysis

Sta-s-cal analysis

Page 12, Lines 58-60: "We examined the rates of comple3on and discrimina3on (i.e. floor/ceiling effects) of indica3ve primary…" – **How? What techniques were used?**

Qualita-ve analysis

Page 13, Line 12: **The authors should have separated the repor-ng of quan-ta-ve and qualita-ve data.**

Results

As I discussed earlier, I recommend the authors report their findings in two separate papers, one for qualita-ve findings and another for quan-ta-ve results. As a result, I will not go into great details with the feedback on this sec-on, as there will be obvious revision of this sec-on.

Lines 48-52: "…with varia3on in student recruitment across FE se[ngs ranging from 41% to 100% at baseline and from 44% to 97% at follow-up." – **I am unsure I understand what this means.**

Line 52: "…par3cipants 95.2% were 16-19 years of age…" – **Who comprised the 4.8%? Conven-onally, the first table in the results sec-on is used to present the sociodemographics of the study sample to help the reader contextualise the results and understand what the study conclusions are based on. I encourage the authors to adopt this approach.**

Line 54: "…30.3% from an ethnic minority…" – **More specificity is needed.**

Discussion

I find the discussion very limited. The study limita-ons are not properly discussed. Obviously, the discussion with evolve hugely based on the recommenda-on of presen-ng the sets of results separately for more logical flow and internal cohesion of the papers.

References

I found a lot of references to be older than ten years. I find it hard to believe that there are no recent cita-ons that the authors could have used in highly researched space as sexuality and HIV/STI preven-on among the youth and young adults. This work would benefit immensely from a literature review exercise with more recent work done in this space.

VERSION 1 - AUTHOR RESPONSE

Reviewer: 1

R1.1 I would like to thank the editor for allowing me to review the manuscript. The paper examined the acceptability of implementing, trialling and estimating the cost of Sexual health and healthy relationships for Further Education (SaFE) intervention. I believe the findings provide additional insights for policymakers and the scientific community. The paper is well written, and readable. However, I believe the authors are required to address a few comments and /or otherwise clarify more to improve the manuscript.

OUR RESPONSE
Thank you for these positive comments, we will endeavour to address the comments and improve the manuscript.

R1.2 In your study population, "FE setting retention was incentivized with a £500 payment on completion of the study." Was this incentive communicated to participants before the intervention started, or only after their participation had ended?

OUR RESPONSE

All FE Settings were informed of the £500 incentive payment at the point of signing up to be involved in the study. Incentives were provided regardless of allocation to intervention/control arms. Participants (i.e., FE staff and students) in the intervention sites would have been unaware of the incentive to their school or college, however, were incentivised to complete the questionnaire by offering them the opportunity to be entered into a competition to win an iPad.

Reviewer 1 comments 3,4,5 and 8 have been grouped together as the responses relate to the fact that we are reporting on a pilot trial not a full-scale trial.

R1.3 (a) How did you control for baseline differences that could arise due to random chance, and the smaller size of the control group, 3 :1?

OUR RESPONSE

We did not control for baseline differences. Given that this was a pilot cluster RCT, the aim of the study was to assess the acceptability of the intervention and trial methods, thus no estimation of effectiveness, that might be affected by imbalance, was conducted. We were not looking at differences between groups as we would do in a full-

scale effectiveness trial. The 3:1 allocation allowed us to collect more information on the feasibility of the intervention delivery than a 1:1 allocation possible given the small scale of the pilot.

R1.3 (b) What measures did you take to address potential confounders resulting from this unequal allocation?

OUR RESPONSE

We stratified randomisation by country (Wales, England) and type of FE setting (sixth form, community college) not to adjust for confounding but to explore the intervention and trial methods in different settings (i.e., country and type of FE setting). We did not estimate effectiveness so did not adjust for any prognostic covariates.

R1.4 You mentioned, "The allocation was stratified by country and type of setting." Was there a strategy in place to prevent contact, including through social media, between students or teachers in the control groups and those in the intervention groups?

OUR RESPONSE

We did not. It is possible some contamination occurred but it would be very difficult to prevent contact between students. We conducted interviews with FE staff and students in control and intervention sites to examine provision of onsite services so would know if anything changed in relation to services provided within control settings over the course of the intervention period.

R1.5 You stated, "It was not possible for study participants (students), teachers, trial managers, the intervention delivery team, or researchers involved in the process evaluation to be blind to intervention status." Considering that teachers and students were aware of their allocation, how did you address the possibility that this awareness could alter their behaviour and potentially influence the outcomes?

OUR RESPONSE: Response bias is always a possibility in an unblinded trial. The effect this has on the treatment effect will need to be considered were the study to progress to a full-scale trial. In this study, baseline data was collected before intervention allocation,

so students were unaware of the allocation of their school at the time of completing baseline questionnaires. At follow-up, data collections were conducted unblinded. At both data collections fieldworkers who were blind to allocation collected data to reduce the effect of students being unblinded.

R1.8 While stratification by country and type of setting helps balance certain factors, what strategies did you employ to assess any residual imbalance within strata or address unmeasured factors?

OUR RESPONSE

None, imbalance was inevitable in some prognostic covariates given there were only eight randomised units. In a full-scale trial, residual imbalance would be expected to be relatively smaller due to the larger number of randomised units, and we would adjust the treatment effect for balancing variables as well as other important prognostic covariates to reduce the effect of imbalance.

R1.6 How did you ensure consistency in the delivery of the intervention across different settings, accounting for variations in training, adherence to the intervention protocol, and contextual factors (e.g., differences in countries and types of settings)?

OUR RESPONSE

The intervention had some standardised inputs but also allowed for some tailoring to the setting and student. The standardised inputs were that the onsite sexual health service is a standard level one service already offered in the NHS therefore would already be standardised. Secondly, an intervention manual / supplementary training materials were provided to school staff to standardise the training. Thirdly, the intervention advertising materials were provided by the study team but in some cases settings developed their own advertising of the onsite sexual health services. The aim of the intervention delivery was to focus on fidelity of function of the intervention (i.e., intervention theory), rather than fidelity of form. Given that this was a pilot, we explored any potential local adaptations of the intervention in our process evaluation to establish whether the intervention was delivered with fidelity.

R1.7 What measures did you take to assess external factors, such as concurrent interventions or changes within the country or setting, that might have occurred during your 12-month intervention period?

OUR RESPONSE: The process evaluation investigated aspects of the context both local to the school, wider community and country that may have had an inhibitory or potentiating effect on the intervention and trial methods. Interviews with FE staff were conducted in both intervention and control sites to examine these factors.

R1.9 Given that your study employed a mixed-methods approach, I think it should be systematically integrated in the way that your qualitative adds something on your quantitative findings ?

OUR RESPONSE: We are grateful for your feedback and have indeed incorporated a mixed methods approach to the pilot cluster RCT. Each of the methods have been selected to address specific research questions as part of the pilot; some including a single (e.g., quantitative) method, and others like the assessment of fidelity included both quantitative and qualitative methods. In places where we have used multiple methods, we have reported on these together (e.g., intervention acceptability and fidelity of intervention implementation).

Reviewer: 2

R2.1 Thank you for the opportunity to contribute positively to the review and betterment of this article. The authors are tackling an important area of research targeting young people's sexual health. Young people are often a group that is difficult to reach and deliver sexual health services to. This study has a potential to answer some of the pertinent questions on how to effectively package these services targeted to this particular group. However, there are important considerations that the authors have to account for before the article can be published, in my view.

I have structured my report into two parts. The first part addresses broad concerns about the article in terms of the structure, methods, and the internal cohesion of the article. The second part highlights the granular issues to be attended to by section.

OUR RESPONSE

Thank you for your comments, we will endeavour to address them in a point by point manner below.

Reviewer comments R2.2, R2.11, R2.23 and R2.24 have been addressed together as they comment on splitting the paper into two separate papers.

R2.2 The title of the article suggests the authors would report about the cluster randomised controlled trial which is a quantitative study. However, the authors also report on the qualitative data they collected during the course of the study. Although it is not a bad idea to report the results of these two approaches, it would be best to report them separately. The challenge with the current article is that both the methods and results of the two study approaches (qualitative and quantitative) are not sufficiently reported. Important details are missing in these two important sections. It would be best for the authors to write two papers, where one paper presents the findings of the qualitative study and another the quantitative results.

R2.11 Lines 56-60: "Structured observations of staff training (n=1 per setting), focus groups with students (n=2 per setting) and telephone interviews with trained FE staff (n=4 per setting) and onsite sexual health service staff (n=1 per setting) examined intervention..." – I encourage the authors to report the two study approaches separately to sufficiently address the methodological considerations of the two study approaches.

R2.23 Page 13, Line 12: The authors should have separated the reporting of quantitative and qualitative data.
R2.24 As I discussed earlier, I recommend the authors report their findings in two separate papers, one for qualitative findings and another for quantitative results. As a result, I will not go into great details with the feedback on this sec-on, as there will be obvious revision of this section.

OUR RESPONSE

We carefully considered how best to report the results of this pilot cluster RCT with embedded process evaluation, which contains both qualitative and quantitative aspects of the study. We decided to combine them into a mixed methods summary framed around the progression criteria as these criteria are the primary outcome of the study. As the progression criteria involve a mixture of qualitative and quantitative methods to assess the combination of both allows us to systematically integrate these data to provide a more comprehensive assessment of the acceptability of the trial methods and intervention.

Reviewer comments R2.3 and R2.10 have been addressed together as they comment on the same thing.

R2.3 It is unclear how long the intervention took before the follow-up measurements were done. The authors only mention the 12-month follow-up. In the text, the authors only clarify that it is 12 months after the baseline. That is not enough, because they do not report on the length of the intervention nor the length of time to follow-up/post-intervention measurements after the implementation of the intervention.

R2.10 Page 7, line 47: "...were taken 12-months post baseline." – What was the time interval between the completion of the intervention and the post-intervention survey? How long after the intervention was the second set of measurements taken?

OUR RESPONSE

We thank you for this feedback and we agree this is not clear. The baseline assessments took place in September/October 2021, intervention delivery was between January and July 2022 and the follow-up assessments were made in September/October 2022. Thus, the 12-month follow-up was 12-months after the baseline assessment not 12-months after the end of the intervention.

Updated Text (Page 7)

...Baseline measures were collected in September & October 2021 and assessed via a student self-report survey prior to randomisation. A second set of measurements were taken 12-months post baseline in September & October 2022, the intervention starting with staff training in November 2021 and the on site services and publication running January to July 2022...

R2.4 Page 5, line 7: "In 2022, NICE recommended..." – The authors should write this acronym in full first before using the acronym.

OUR RESPONSE

This has been amended in the updated document. Amended text (Page 5):

...In 2022, National Institute for Health and Care Excellence (NICE) recommended that sexual health services should be considered in non-clinical settings...

R2.5 Line 20: "...settings have a transient student population,..." – In what way?

OUR RESPONSE

Unlike 6th forms attached to schools (where students may have already studied for 5 years, and then continue for another two years in their post 16 education), in FE colleges in the UK, students will typically only study for two years. Students in colleges tend to have more flexible timetables where they only attend on campus at certain times of the day or week for certain classes (as opposed to a full, five day, 9am-3pm school-day structure in secondary school). Similarly, historically, FE settings catered for more applied or vocational programmes of study, which involve more placements; students may be on campus for two weeks to study, then on placement for several weeks. All of this contributes to the transient nature of UK FE settings. Amended text (Page 5):

...However, FE settings have a transient student population with flexible timetables and attendance is only needed on campus at certain times of the day or week, sites also vary considerably in size as well as programmes and services offered. This...

R2.6 Line 22: "...as range of programmes and services offered." – The authors should provide a source(s).

OUR RESPONSE

A suitable source for this statement is the Association of Colleges (https://www.aoc.co.uk/). This is now referenced in the paper.

R2.7 Page 6, line 6: "Study design and participants" – More description of the study design is needed.

OUR RESPONSE

As requested, we have added more description of the study design to the methods section. We also cite our protocol which contains an in-depth description of the study design. Here is the additional text (Page 6):

> "This study involved a two-arm repeated cross-sectional pilot cluster randomised controlled trial (cRCT) of SaFE compared to usual practice, including a process evaluation and an economic assessment. The full details of the study design can be found in our published protocol elsewhere.[24]"

R2.8 Line 10: "…extension for pilot and feasibility trials." – This point needs to be made explicitly. It is unclear that the study was a pilot until later.

OUR RESPONSE

We thank you for the opportunity to clarify this. As above (R2.7) we have added in the statements (Page 6):

"This study involved a two-arm repeated cross-sectional pilot cluster randomised controlled trial (cRCT) of SaFE compared to usual practice, including a process evaluation and an economic assessment. The full details of the study design can be found in our published protocol elsewhere.[24]". We have also added the word 'pilot' into the subsequent statement for clarity "This pilot study was reported in accordance with the CONSORT extension for pilot and feasibility trials. [25]". That the study is a pilot cRCT is also mentioned in the title and abstract.

R2.9 Lines 12-14: "…and all interested were visited to discuss the study in more detail and agree a research contract." – To what extent did this approach introduce bias to this study?

OUR RESPONSE

Visiting educational settings to recruit them to take part in research is standard procedure in all cluster RCTs. We are unsure what bias the reviewer is referring to, but do not believe that this approach would have introduced any undue bias into the study.

R2.12 Page 8, lines 14-16: “Students were provided with information sheets and consent forms, and, following informed consent,...” – It is unclear to me how consent was sought among students younger than 18 years old. Was the parents' approval sought for these students?

OUR RESPONSE

Informed consent was provided by all participants. Parental approval was not required as students were all of an age where they were deemed to have capacity to provide informed consent themselves (i.e., older than 16). Schools and colleges were offered the option of writing to inform the parents so that parental opt-out could be gained if they had particular reason to exclude their child however all settings declined to offer this and sought informed consent directly from students.

R2.13 Line 18: “...questionnaire were offered entry into a prize draw to win an iPad.” – I am wondering if this approach was not seen as an undue inducement.

OUR RESPONSE

This incentive to encourage questionnaire completion is widely used within clinical trials and is accepted by ethical committees. In the case of this study both NHS and School ethical review boards found offering this incentive acceptable.

R2.14 Lines 18-20: “The baseline survey occurred in Sep/Oct 2021 and follow-up 12 months post baseline (Sep/Oct 2022).” – This statement is not part of ethics discussion.

OUR RESPONSE

We thank you for this feedback, we have removed this statement from the ethics section and further detail has been added to the data collection section in line with comment R2.10, amended text on page 7 as follows:

...Baseline measures were collected in September & October 2021 and assessed via a student self-report survey prior to randomisation. A second set of measurements were taken 12-months post baseline in September & October 2022, the intervention starting with staff training in November 2021 and the on site services and publication running January to July 2022...

R2.15 Line 22: "Trained fieldworkers attended social areas and lessons to invite students to participate." – Is this not part of the recruitment strategy?

OUR RESPONSE

Thank you for this feedback, we have moved this statement to the data collection section.

R2.16 Line 24: "Ethical approval was..." – Is it not a requirement in this context to obtain gatekeeper permission from the participating institutions?

OUR RESPONSE

We thank for you for the opportunity to clarify here; we have added in the following statement. Updated text (Page 8):

...FE setting gatekeeper permission was sought from each participating institution; college managers, head teachers or deputy head teachers each signed a memorandum of understanding to agree to take part in the research...

R2.17 Line 60: "...4Ps..." – The authors should discuss this (4Ps) further. What do these 4Ps represent?

OUR RESPONSE

We have described the 4Ps below the statement however we agree this could be clearer. It will now read (Page 9):

...Social marketing principles were used to address the "4Ps" selling consumers (students) a **P**roduct they want (sexual health and relationship services) in an accessible

Place (their FE setting) at a low **P**rice (free) with **P**romotion (via staff and publicity materials).[28] ...

R2.18 Page 9, Line 5: "...at low price..." – Grammar? Should this not read, "... at a low price..."?

OUR RESPONSE

This has been amended in the updated document.

R2.19 Page 11, Lines 11-15: "This study builds on 15 months of work with over 2000 students and 200 staff from six FE settings, 12 sexual health staff and an advisory group of 16-21 year olds (ALPHA) to explore which components should be combined into an intervention." – Does this mean that all these individuals were part of this study?

OUR RESPONSE

This is a reference to a previously reported study from which this pilot trial follows on. The individuals mentioned were part of the previous study and not the one reported in the paper.

Updated in page 11:

This study builds on 15 months of previously published work with over 2000 students and 200 staff from six FE settings, 12 sexual health staff and an advisory group of 16-21 year olds (ALPHA) to explore which components should be combined into an intervention.[24] We discussed the findings, intervention and methods for this project with 30 stakeholders at a consultation event.

R2.20 Lines 33-35: "...of the pilot cRCT was whether progression to a phase III RCT is justified in terms of progression criteria..." – The authors need to explicitly mention the design of this study earlier. It is not discussed explicitly in the methods section.

OUR RESPONSE

As above, we have now stated in the design section "This study involved a two-arm repeated cross-sectional pilot cluster randomised controlled trial (cRCT) of SaFE compared to usual practice, including a process evaluation and an economic assessment. The full details of the study design can be found in our published protocol elsewhere.[24] "

R2.21 Line 44: "We also examined the indicative primary outcomes of a future phase III trial." – It seems like this part is outside the scope of the study. Relevance?

OUR RESPONSE

Assessing the distribution, variability, and level of missing data is an important part of the assessment of the acceptability of an indicative primary outcome measure. If levels of missing data are high or there is evidence of floor or ceiling effects these factors need to be taken into account either in the choice of another suitable outcome measure, or to inflate the sample size calculation if a subsequent phase III trial is warranted.

R2.22 Page 12, Lines 58-60: "We examined the rates of completion and discrimination (i.e. floor/ceiling effects) of indicative primary..." – How? What techniques were used?

OUR RESPONSE

eTable 2 and 3 show the percentage of missing data for binary outcomes. For continuous outcomes, means, medians, minimums and maximums were described to allow an assessment of floor and ceiling effects.

R2.25 Lines 48-52: "...with variation in student recruitment across FE settings ranging from 41% to 100% at baseline and from 44% to 97% at follow-up." – I am unsure I understand what this means.

OUR RESPONSE

To clarify, across the eight sites, at baseline student recruitment to the survey ranged from 41% to 100% response rates, and at follow up, across the eight sites, recruitment ranged from 44% to 97% response rates to the survey. We have clarified this with the amended text below (Page 14):

...with variation in student recruitment to take part in the survey across FE settings ranging from 41% to 100% at baseline and from 44% to 97% at follow-up...

R2.26 Line 52: "...participants 95.2% were 16-19 years of age..." – Who comprised the 4.8%? Conventionally, the first table in the results section is used to present the sociodemographics of the study sample to help the reader contextualise the results and understand what the study conclusions are based on. I encourage the authors to adopt this approach.

OUR RESPONSE

eTable 1 in the supplementary materials shows the baseline characteristics in the overall sample and by arm.

R2.27 Line 54: "...30.3% from an ethnic minority..." – More specificity is needed.

OUR RESPONSE

We agree. Ethnic minorities comprised of mixed or multiple ethnicities, Pakistani, Indian, Bangladeshi, Chinese, African, Caribbean or Black, and Arab. Text has been updated (Page 14):

... ethnic minority comprising of; Mixed or Multiple ethnicities, Pakistani, Indian, Bangladeshi, Chinese, African, Caribbean or Black, and Arab. 96.5% lived with a parent...

R2.28 I find the discussion very limited. The study limitations are not properly discussed. Obviously, the discussion with evolve hugely based on the recommendation of presenting the sets of results separately for more logical flow and internal cohesion of the papers.

OUR RESPONSE

We have responded to the reviewer's comment about the separation of the paper into two, but would welcome the opportunity to expand on the discussion of limitations. We have added in page 26.

The timing of the research meant that the study had to be put on hold for a period during the COVID-19 pandemic and while restarting in January 2021, there was significant uncertainty about the conduct of school-based research, and the delivery of sexual health services. During this period the study was impacted by staff and student sickness rates across both FE and service delivery teams, as well as impacts on student and staff attendance, and freedom to move throughout FE sites. Similarly, COVID-19 pandemic restrictions at FE settings meant the intervention was not implemented for as long as planned (up to 23 weeks vs 39 weeks). In combination, this has adversely impacted the provision of the sexual health service, attendance at the service, as well as engagement of students with the completion of the baseline and follow-up surveys.

The study did not meet the 'green' threshold for one progression criterion relating student recruitment whereby 60% of students approached to participate should have agreed to do so; in this case 57% of students approached agreed to take part. COVID-19 and social/physical distancing may have impacted students' engagement with the survey, and fewer students had returned to campus during the study, however overall higher response rates were identified at baseline (61.1%; 1131/1852 students) compared to at 12-month follow-up (53.9%; 1181/2193 students). Before moving to a full-scale trial, the study team needs to work closely with young people to develop data collection methods and bespoke approaches to increase student engagement with the survey and increase the number of students consenting when approached to take part.

In line with previous research in FE settings,[27] completion of the staff logbooks was very low across intervention sites. However, it is possible that in the case of the SaFE intervention, this was compounded by the additional administrative burdens placed on teachers during the COVID-19 pandemic. More work needs to be done with FE teachers to understand their competing demands and to explore how best to

encourage and perhaps incentivise completion of logbooks in the event of student intervention relating to sexual health and dating and relationship and/or gender-based violence.

Finally, methodologically, qualitative interviews with FE students were undertaken with self-selecting samples. Students were invited to take part on a voluntary basis and were often invited to take part through their teachers, which may have impacted on the representativeness of the data collected.

R2.29 I found a lot of references to be older than ten years. I find it hard to believe that there are no recent citations that the authors could have used in highly researched space as sexuality and HIV/STI prevention among the youth and young adults. This work would benefit immensely from a literature review exercise with more recent work done in this space.

OUR RESPONSE: We agree. Where possible we have now incorporated more recently published papers into the manuscript. Others which may be older but either more recent data do not exist or they are seminal papers in the field. Here are some of the more recently published papers we have now included at the reviewers request:

Blake, C. , Lewis, R. , Riddell, J. , Willis, M. , Wylie, L., Dawson, K., Moore, L. and Mitchell, K. R. (2024) Prevention of, and response to, sexual harassment at secondary school: a system map. Social Science and Medicine, 358, 117092. (doi: 10.1016/j.socscimed.2024.117092)

Marcantonio TL, Weese J, Willis M. Rates of Forced Sexual Experiences Among High School Students From 2001 to 2019. Journal of Interpersonal Violence 2022;37:21-22

VERSION 2 - REVIEW

Reviewer 1

Name Habtu, Yitagesu

Affiliation Addis Ababa University, School of Public health

Date 18-Oct-2024

COI

Authors have clarified, and addressed all my issues.
